# Skilled we-intentionality: Situating joint action in the living environment

**DOI:** 10.12688/openreseurope.13411.2

**Published:** 2021-09-27

**Authors:** Julian Kiverstein, Erik Rietveld

**Affiliations:** 1 Psychiatry, Academic Medical Center of the University of Amsterdam, Amsterdam, 1105AZ, The Netherlands; 2 Philosophy, University of Twente, Enschede, The Netherlands; 3 Institute of Logic, Language and Computation, Humanities, University of Amsterdam, Amsterdam, The Netherlands

**Keywords:** Joint action; we intentionality; skilled intentionality; affordances; dynamical systems theory; embodied cognition; philosophy of action

## Abstract

There is a difference between the activities of two or more individuals that are performed jointly such as playing music in a band or dancing as a couple, and performing these same activities alone. This difference is sometimes captured by appealing to shared or joint intentions that allow individuals to coordinate what they do over space and time. In what follows we will use the terminology of we-intentionality to refer to what individuals do when they engage in group ways of thinking, feeling and acting. Our aim in this paper is to argue that we-intentionality is best understood in relation to a shared living environment in which acting individuals are situated. By the “living environment” we mean to refer to places and everyday situations in which humans act. These places and situations are simultaneously social, cultural, material and natural. We will use the term “affordance” to refer to the possibilities for action the living environment furnishes. Affordances form and are maintained over time through the activities people repeatedly engage in the living environment. We will show how we-intentionality is best understood in relation to the affordances of the living environmentand by taking into account the skills people have to engage with these affordances. For this reason we coin the term ‘skilled we-intentionality’ to characterize the intentionality characteristic of group ways of acting, feeling and thinking.

## Plain language summary

How could the environment be structured to allow people to act as groups?

People act together all the time. Think of a couple painting their house together or carrying a heavy object like a piano up a steep flight of stairs in an Amsterdam apartment. These are the kind of activities we aim to understand in this philosophical article. In philosophy researchers have written a good deal about how it is possible for two or more agents to think or act as members of a group. To carry the piano up the stairs, for instance, people must avoid getting in each other's way or pulling in different directions.

Our research group investigates how people structure the environment in ways the support their actions. In this article our aim is to show how the environment also has a crucial role to play when people do things together. The stairs and the weight of the piano for instance tightly constrain how removal men move their bodies as they heave the piano up the stairs. The main contribution of this article is to describe the much overlooked reliance agents have on the structure in the environment when they act together.

## Introduction

People take part in collaborative and joint activities. They help each other to move house. They play games against each other and make music together. They act in solidarity with each other to protest against social injustice. In each of these examples people are doing things together; they are acting jointly. Joint actions are different from co-actions in which multiple individuals each do their own thing. Philosophers have accounted for this difference by appealing to a special class of intentional states referred to variously as we-intentions, shared-intentions, or joint-intentions ( Bratman, 1993; Butterfill, 2012; Gilbert, 1989; Gilbert, 1996; Pacherie, 2007; Sellars, 1963; Searle, 1990; Tuomela & Miller, 1988; Tuomela, 2005). In what follows we will use the terminology of ’we-intentionality’ to refer to what individuals do when they think, act, or feel as a member of a group. We take thinking, acting and feeling to be interrelated dimensions of skilled action ( Dewey, 1934; cf. Rietveld *et al*., 2018). We-intentionality, as we will use this term, is a characteristic of skilled actions performed collaboratively and collectively with other agents. Our aim in this paper is to argue that we-intentionality is best understood in relation to a shared living environment in which acting individuals are situated. By the ‘living environment’ we mean to refer to places and everyday situations in which humans act. These places and situations are simultaneously social, cultural, material, and natural. We will use the term 'affordance’ to refer to the possibilities for action the living environment furnishes ( Gibson, 1979/2014). Affordances form and are maintained over time through the activities people repeatedly perform in the living environment ( Kiverstein *et al.*, 2019; Rietveld & Kiverstein, 2014; Van Dijk & Rietveld, 2017). We-intentionality is, we claim, best understood in relation to the affordances of the living environment and by taking into account the skills people have to engage with these affordances.

Philosophers and cognitive psychologists have typically approached we-intentionality by asking what it takes for individuals to perform an action together and to share an intention or goal to do so. Actions are the doings of agents, they are not passively undergone by agents. It is the person’s intention that is standardly taken to distinguish an action of an agent from an accident for which the agent is excused. Some of the actions that agents can control are joint actions two or more agents perform together. Philosophers have suggested that what is distinctive of these joint actions is that they are caused by intentions and goals that are shared among the individuals participating in the action. The philosophical debate has been concerned with the conditions under which two or more individuals can be said to share an intention or goal.

However, the focus on this question has led philosophers to neglect the contribution of the living environment to we-intentionality. Infants grow-up in an environment structured by caregivers to facilitate the infant’s development of skills for participating in the daily routines of their community. They learn about what can be done with the things around them, in part through interaction with other people in structured social settings ( Reed, 1996, ch.9 & 10). The ecological psychologist Edward Reed described how other people create what he called a “field of promoted action” for infants ( Reed, 1991) ^1^. What is promoted to the infant are everyday action possibilities that allow the infant to understand, develop skills, and eventually take part in the practices of their community. The education of the child’s attentions to those affordances results in joint attention (also see Segundo-Ortin & Satne, forthcoming). Part of the experience the child and caregiver undergo is that they are together attending to a possibility for action. What the child learns in the process is that affordances are not only for individuals but allow for the pursuit of joint activities such as cooking, tidying, playing, or making things together.

Drawing upon these important insights from the research tradition of ecological psychology, we will provide an account of we-intentionality in terms of a skilled agent’s selective responsiveness to relevant affordances, or what we propose to call *skilled we-intentionality*. When people engage in activities together, we will argue, it is the shared relevant affordances of the living environment that explain how they manage to coordinate with each other. In acting together, agents dynamically couple to each other in ways that are constrained and scaffolded by the affordances of shared relevance to them.

Our paper consists of four sections. In section one, we introduce the central idea of our paper that we-intentionality should be understood in relation to the affordances of the living environment. In section two we introduce the concept of skilled intentionality as selective responsiveness to multiple relevant affordances. In section three we show how, when people act jointly, they are able to make their actions coordinate with each other over space and time because they are responsive to affordances of shared relevance. In section four we explain what it is for affordances to be of shared relevance to multiple individuals. We conclude that group modes of thinking and acting are made possible by affordances of the living environment that are of shared relevance to multiple individuals. 

## The role of affordances in action coordination

We-intentionality is standardly taken to be a property of the mental states that account for how individuals can think, act and feel as members of a group or communal practice ^2^. We will use the term to refer to what agents do when they skilfully engage in and contribute to group and communal activities such as playing music in a band, taking part in a team sport, working together on a collaborative project, or acting in agreement with a local custom. These activities require skill and sensitivity on the part of the agent. The agent must sensitively adapt and adjust what they do when they take part in group or communal activities so that it fits with the demands of the particular circumstances in which they are acting. They must be sensitive to whether their performance is unsatisfactory or adequate, appropriate or inappropriate relative to what others in the group expect of them ^3^.

The term ‘we-intentionality’ can be applied at a variety of scales from joint action at the scale of the dyad, to collective patterns of skilled action at the scale of groups and communities. The conceptual framework for understanding we-intentionality we propose is intended to generalise across these scales. This is reflected in the examples we have chosen, some of which are examples of dyads performing activities together. Other examples concern communal activities in which individuals regulate their actions so as to make them conform with the patterned practices of their communities. Our aims in this paper are thus general. We propose a conceptual framework – the Skilled Intentionality Framework ( Rietveld *et al.*, 2018) – for making sense of we-intentionality. The task of applying this framework to the rich variety of phenomena philosophers have discussed under the heading of we-intentionality goes beyond what we can achieve in this paper.

We propose to use the term ‘we-intentionality’ in this *scaleable*, and therefore slightly non-standard way, in part on developmental grounds. Shared ways of thinking, feeling and acting can already be observed in dyadic engagement between infants and their caregivers in family life ( Hobson, 2002; Reddy, 2018; Rochat & Robbins, 2017; Rakoczy, 2017; Vincini & Gallagher, 2021; Satne & Sallice, 2020; Segundo-Ortin & Satne, forthcoming). The collective acceptance of the rules and norms of communal social practices and cultural institutions and conventions may build upon the ability to coordinate and attune to the actions and reactions of others that emerge in early development. Think for instance of emotional tuning to other’s approval or disapproval that underpin a child’s developing a sense of the customs and norms of her community ( Haugeland, 1990; Satne, 2014). Our adult abilities to make sense of complex social institutions such as governments, universities, and corporations builds upon and grows out of abilities to blindly, but nevertheless sensitively, follow shared norms that emerge very early in development.

As noted in our introduction, many human skills are mastered by children not by themselves but in social interaction with their caregivers. Many of the problems the infant runs into in skill development are thrust upon the infant by its caregivers ( Reed & Bril, 1996). The caregiver calls the attention of the infant to particular affordances provided by their living environment. Think for instance of the motor problems the infant encounters in trying to handle eating implements such as spoons or chopsticks to feed themselves while eating with their family. The motor problems in this case originate in the skills the child needs to develop for engaging in a communal activity of eating together. These skills make possible what we are calling we-intentionality defined as what agents do when they engage in and contribute to a group or communal activity. The motor problems the infant encounters have a particular form because of the multiple affordances it needs to coordinate with to take part in a family meal. In the process of being introduced and promoted to the child by its caregivers, these affordances become joint affordances that will eventually enable the child to take part in this shared activity. This is a simple illustration of the central claim of our paper that we-intentionality should be understood in relation to the affordances of the living environment. 

The living environment has been largely absent from the philosophical discussion of we-intentionality ^4^. The debate surrounding the nature of we-intentionality has been mostly focused on the question of whether we-intentions are reducible to complex aggregates of interlocking attitudes of individual agents. Searle (1990) for example, argued against such a reduction of we-intentions to the intentions of individuals. For Searle we-intentions are distinguished by a psychological mode “that must make reference to collectives” (1990: p.408). Bratman (1993) by contrast denies that we-intentions form a special, psychologically primitive class of intentions. He has provided a reductive account of what he calls “shared-intentions” in terms of the interlocking intentions and attitudes of the participants in a joint action. It is the interrelatedness of the individual’s intentions and the complementarity of their plans that Bratman takes to be explanatory of joint actions. We-intentions can be distinguished from the intentions of individuals because they require there to be certain relations between the intentions and attitudes of individuals and for the individuals to know these relations obtain. 

Bratman’s account is tailor-made for cases of conscious and deliberate action in which agents act in pursuit of some shared plan formed prior to the execution of a joint action. Plans are conceived by Bratman as supporting the coordination of various activities over long periods of time. The formation of such a long-term plan requires practical reasoning to work out the course of action that is conducive to bringing about this plan. It may well require the formation of a hierarchy of sub-plans and intentions for bringing about each of these sub-plans. The account of interlocking intentions Bratman provides is perhaps well-suited to account for the negotiations that might unfold between people when they plan to perform an action together (e.g., Bratman, 2014). His account does not aim to explain how multiple agents succeed in putting the plans they have made into effect, coordinating what they are doing when they act together in a particular situation.

Searle’s account of we-intentions also has nothing to say about how the actions of different individuals must fit together in order for them to successfully perform a joint action. Searle seem to think the answer to this question can be given by appeal to biological background capacities that are not themselves intentional phenomena ( Searle, 1995, ch.6). However, this division of labour between philosophy and science will not do. Searle’s distinction between we-intentions and non-intentional background capacities introduces a problematic divide within the mind between ‘lower-level’ mechanical coordinative structures and ‘higher-level’ we-intentions and goals. The details of how a we-intention is implemented by multiple individuals as they act together in a particular situation are treated by Searle as a matter of the causal capacities of the brain that enables, we-intentional states ( Searle, 1995: p.129). We question whether this causal separation of ‘lower-level’ coordinative processes from ‘higher-level’ we-intentions can be made in practice (cf. Pacherie, 2007). 

Butterfill (2012) correctly rejects Searle’s separation of we-intentions from non-intentional background capacities when he describes how coordinative structures form across two or more bodies through the coupling of perception and action (cf. Richardson *et al.*, 2009; Shockley *et al.*, 2009; Tollefsen & Dale, 2012). He argues for a class of plural activities that require only a shared goal or outcome. A group of ants for instance can work together to kill a large insect. The ant’s individual behaviours are organised to bring about the death of the insect but they have this effect only through their coordination. Butterfill introduces the concept of a shared goal to explain how the actions of the individual ants could be coordinated. Their actions can be directed at one and the same goal as a consequence of what Butterfill calls ’emergent coordination’. Coordination is the outcome of perception-action couplings between agents and does not require the agents to form plans or to reason about each other’s intentions. Multiple agents can begin to act together because the actions of each of them are responsive to the same environmental cues and motor routines, and each agent makes more or less the same contribution to bringing about a shared goal. 

As an example of emergent coordination consider the following experiment in which participants were given the task of lifting planks from a moving conveyor belt ( Richardson *et al.*, 2007; Richardson *et al.*, 2010). Participants were told that they were only allowed to lift the planks by their ends, not from the centre which meant that some of the planks were too long to be lifted alone, and had to be carried by two participants working together. The experimenters found that people switched from lifting the planks alone, to working as a dyad based on the ratio of their arm span to the plank length ( Richardson *et al.*, 2007: 849). As the planks got closer to an individual’s maximum arm span, so they spontaneously switched from moving the plank alone to moving the plank together with the other agent. The presence of the other person in this case induces emergent coordination. In the presence of the other person the plank becomes “jointly liftable” or “liftable by us” ( Knoblich *et al.*, 2011; cf. Abramova & Slors, 2015: p.528–9). The possibility to lift the long plank is an affordance for the dyad comprised of the two co-actors when they work together.

There are two important points we wish to highlight from this example of emergent coordination. Firstly, what Butterfill calls the ‘shared goals’ of the individuals should be understood in relation to the affordances of the plank ( Abramova & Slors, 2015). Recall that one of the questions that has occupied philosophers working on we-intentionality is how the intentions of distinct individuals can be coordinated over space and time so that the individuals can take part in a joint action. Reductive accounts like that of Bratman have supposed that we-intentions must somehow represent who will do what and when and that such a representation must be common knowledge among the agents taking part in the joint action. However, it is less clear that this kind of complex “mental gymnastics” ( Chemero, 2009) is necessary once we think of intentions in relations to the affordances of the environment. We expand on this point in the next section.

Secondly, when the two individuals work together to the lift the plank they can be thought of as forming an interpersonal synergy. The notion of synergy here is that of a constraint that when applied to a system causes the elements of a system to form units that work together. Chemero (2015) gives as an example of a synergy laser in which “large amounts of energy constrain photons so that they coordinate their behaviour with another over long spatial and temporal scales” (p.143). The affordance of liftability can be thought of as a constraint that enables the two individuals to temporarily form an interpersonal synergy and act together as a single unit. We return to this point in section three. Once we keep these two points in mind, the problem of how two or more individuals can act together looks very different than what we saw above in for example, the accounts of Searle and Bratman. Coordination is something that unfolds out in the open between them, instead of being something that can only be achieved through complex reasoning about each other’s mental states. 

## The Skilled Intentionality Framework: explaining goals and intentions

Our aim in this section is to show how the work that shared goals and intentions are asked to do in philosophical accounts of joint action can instead be performed by affordances of shared relevance. We provide an analysis of intentions and goals in terms of what we will call ‘skilled-intentionality’ ( Bruineberg & Rietveld, 2014; Kiverstein & Rietveld, 2021; Rietveld *et al.*, 2018; Van Dijk & Rietveld, 2017). The concept of skilled intentionality is a generalisation and elaboration of what Merleau-Ponty (1945/2002) called ’motor intentionality’ to apply to skilled expertise in general. Merleau-Ponty talks about the skilled typist for instance as having knowledge in her hands. Based on her motor skills she experiences the keyboard as inviting certain movements of her fingers as she types (1945/2002: 166). Similarly, the goalkeeper’s football skills allow them to time their dive just right, so as to intercept a ball heading towards their goal.

Merleau-Ponty describes the actions of the skilled agent as being drawn forth from them by the world. The football field is for instance “pervaded by lines of force (the ‘yard’ lines; those which demarcate the ‘penalty area’) … which call for a certain mode of action.” ( Merleau-Ponty, 1943/1962: 168) We’ve used the different terminology of the ’field of relevant affordances’ to capture the same idea of skilled actions being solicited or invited by the world ^5^. ’Relevant affordances’ are the possibilities for action offered by the environment that ’stand-out’ in the sense of being marked out as salient or significant from the other affordances the living environment provides (cf. Withagen *et al.*, 2012). If I am currently hungry for instance, the apple that is sitting by my computer will appear more alluring than when I have just eaten.

We will use the term the ‘landscape of affordances’ to refer to the totality of affordances that can be found in the ecological niche of a given form of life. The landscape of affordances is exceptionally rich in terms of the possible actions it offers. Yet individuals are typically ready to respond selectively to affordances in ways that are appropriate to the context, and that reflect their own internal states that arise in relation to their situation. How is it possible that individuals are only responsive to relevant affordances in each particular situation? The very same affordances can stand out as relevant to an individual in one situation, while in a different situation the same affordance does not move them at all. What accounts for this difference?

We do not think appealing to the agent’s goals and intentions helps to answer this question ( Van Dijk & Rietveld, 2020). It just invites the question: why does the agent have the goals and intentions that they do? We thus agree with Lucy Suchman when she writes:

To characterise purposeful action as in accord with plans and goals is just to say again that it is purposeful and that *somehow*, in a way not addressed by the characterisation itself, we constrain and direct our actions…How we do that is the outstanding problem. Plans and goals do not provide the solution for that problem, they simply restate it. ( Suchman, 1987: 47–8, cited by Heft, 2001: 311)

Rather than presupposing that the agent has pre-existing plans, intentions and goals, we seek an account of goals and intentions in terms of the self-organising dynamics of the agent situated in a landscape of affordances. A system is self-organising if it exhibits ordered and regular patterns of macroscopic behaviour as a result of the endogenously generated interactions among its component parts, without any centralised control ( Bruineberg & Rietveld, 2014; Freeman, 2000; Kelso, 1995; Ladyman & Wiesner, 2020; Strogatz, 1994; Tschacher & Haken, 2007; Tsuda, 2001). The self-organising system that we take to be explanatory of skilled intentionality is the whole animal-environment system. 

The animal’s responsiveness to affordances can be described in terms of an attractor landscape ^6^ with a particular form determined by the animal’s learning history and the skills and abilities it has developed over the course of this history. Large scale patterns of activity spontaneously form in the brain and the rest of the body as a consequence of a history of engagement in a skilled activity. Certain patterns of activity are selected and preserved because they have proven to be practically useful to the organism in the past for coordinating with affordances ( Reed, 1996). The organism’s moves towards a basin of attraction as it prepares to act. Each basin of attraction corresponds to an affordance-related state of action readiness.

The affordances that stand out as relevant to an agent are those that are sensed as affectively significant because they contribute in some way to improving its grip. We borrow the notion of grip from Merleau-Ponty (1945/2002) to refer to the way the skilled individual is ready to move so as to improve its relation to the environment. Think of turning the dial on a microscope to get a better view of a biological specimen as an example of tending towards an optimal grip. So long as the specimen looks blurry through the microscope one feels one is not quite seeing it right. Based on this feeling one turns the dial until the image one is seeing through the lens is improved (i.e., sharper, and better defined). To be a skilled agent is to be able to maintain a good grip on the multiple relevant affordances required for the performance of a skilled action. The strikers in a football team will be sensitive to the events that are playing out on the football pitch because of their years of practice in the game, ready to take advantage of opportunities to strike on goal when they arise. Any change in the game or in the body can be sensed as disattunement, a deviation away from good grip, which, if all goes well, the striker will be affectively moved to reduce thereby achieving a better grip.

The states of bodily action readiness that relevant affordances elicit are inherently affective ^7^. The agent in acting skilfully so as to tend towards an optimal grip modifies his or her relation to the world in a way that is in line with what matters to them ^8^. If all goes well the animal will not come to rest in any basin of attraction but will be ready to switch to another attractor if a course of action does not proceed as anticipated ( Bruineberg & Rietveld, 2019; Dreyfus, 2007; Freeman, 1987; Freeman, 2000). In this way the skilled agent doesn’t just tend towards a single attractor basin at any given moment but is simultaneously influenced by multiple attractors. They are ready to transit between multiple attractors and can therefore be ready to act on multiple affordances simultaneously.

Now that we have the concept of skilled intentionality in place, let us consider again the debate between Searle, Bratman and others we discussed in the previous section concerning the reducibility or otherwise of we-intentionality to the interlocking mental states of individuals. What is in question in this debate is the possibility of explaining we-intentionality in terms that do not presuppose the sharing of mental states. In the Skilled Intentionality Framework, it is the shared affordances of the living environment that are appealed to in making sense of the coordination of the activities of multiple individuals. The sharing of affordances come in in two ways in our account. First, the landscape of affordances is defined in relation to a *shared* form of life. The affordances that the landscape makes available have formed through patterns of practice that are constituted by the coordinated behaviour of multiple individuals. Individuals and collectives of the given form of life are situated in the same landscape of affordances; they share the same landscape of affordances. 

Second, interpersonal synergies form on the basis of affordances of *shared* relevance, as we will see in more detail in the next section. The abilities that are pooled are abilities for distinguishing better from worse, adequate from inadequate, appropriate from inappropriate, or correct from incorrect in the context of a particular situation in a given sociomaterial practice ^9^. We use the term ‘situated normativity’ to refer to this dimension of skilled action ( Rietveld, 2008; van den Herik & Rietveld, 2021). It is based on the pooling of abilities for making these kinds of normative distinctions that individuals are able to tend towards an improved grip on a situation in acting together as a group. Crucially however, the account we provide of *skilled we-intentionality* does not make appeal to a *sui generis* or presupposed type of shared or collective mental state. This is because all cases of skilled intentionality, as we show in the next section, require coordinating with other members of a practice. Skilled intentionality is always already taking into account the established sociomaterial practices because affordances owe their existence in part to a shared social, cultural, material and natural form of life/living environment. Furthermore, the situated sensitivity to doing better or worse we refer to as ‘situated normativity’ is developed by taking part in practices.

## Coordinating with others in a shared landscape of affordances

As we started out noting in section one, many of the skilled activities humans engage in are learned from others in their community. The affordances that invite an individual to act do so because of the individual’s history of engaging in those practices, which gives them a sense for what it is appropriate to do in a particular situation. Inviting affordances thus contribute, albeit on a small scale, to the continuation of a wider practice. As an example of this dynamic consider how in the Netherlands, the trains used for long journeys will often have a few carriages in which travellers are expected to remain silent. The passengers sitting in a silent area can use their time on the train to work, enjoy reading a book, or listening to music without disturbance from other passengers. The continued availability of such affordances however depends on what train passengers do when they sit in such a place on the trains. Each passenger can make a small contribution to the continuation of this practice by refraining from answering their phone, or from entering into conversation with their fellow travellers. Their responding to this invitation contributes to the continuation of the affordances of this place. If each person were not to play their part, there would soon cease to be any difference between silent carriages and any other seat on the train ( Van Dijk & Rietveld, 2017).

Whenever an individual responds appropriately to an inviting affordance in ways that are laid down in an ongoing practice, like in the example of the silent carriage, they are also coordinating with other members of this practice. The contribution to the continuation of the wider practice of silent areas is an example of action coordination albeit over longer time scales. An individual’s participation in joint action can be compared with an individual’s adequate participation in large-scale sociomaterial practices ^10^. What these activities (acting jointly and acting in agreement with a standing practice) share in common is a sensitivity to doing better or worse in the particular situation in one’s engagement with affordances. We call the multiple affordances that stand out as inviting the ‘field of relevant affordances’ ( Bruineberg & Rietveld, 2014; Rietveld & Kiverstein, 2014). The affordances that belong to the field are those that contribute to improving an agent’s grip on a situation. What counts as improvement in grip is however relative to one’s sense of what actions the situation demands. In the silent train carriage for instance, receiving a phone call will invite declining if one is familiar with the customs of travelling by train in the Netherlands. For a tourist unfamiliar with these customs, a ringing phone will simply invite answering. The individual agent, in acting to improve grip, is acting based on their sense of what others in their social group or socio-cultural practice would do. We are suggesting that over time, this can be thought of in terms of acting in coordination with this socio-cultural practice or social group. What happens over this longer time scale isn’t so different from what happens when individuals act together in the here and now.

So far, we have been concerned with the contribution of a field of relevant affordances to we-intentionality. In the next section we turn to the notion of an interpersonal synergy. We will see below that interpersonal synergies form through engagement with affordances of shared relevance. Joint actions within a dyad can also be thought of as a situated self-organising process of selectively responding to multiple relevant affordances at the same time. When the two individuals cooperate in the plank lifting experiment, they do so by adapting and adjusting their behaviour to each other and to the affordances of the plank. Importantly, Kerry Marsh (one of the experimenters involved in carrying out the original study) has compared this switch to what an individual does when she shifts from lifting an object with one hand to lifting a larger object with two hands ( Marsh, 2015: 317–8). The process of switching from removing the plank as an individual agent to doing it together should be seen as a self-organising process that arises spontaneously, based on the dynamic coupling of the agents with the field of relevant affordances. It is in this respect no different from the spontaneous switching when lifting an object with one hand, to lifting the object with two hands that happens within a subject based on the dynamics of the agent-environment coupling.

## Interpersonal synergies

People coordinate and join forces in acting together by responding to affordances of shared relevance in the living environment. When an affordance is of shared relevance to two or more individuals, these agents will then be ready to act together, to cooperate with each other and to pool their individual abilities, so as to improve grip on the overall situation in which the affordances are nested. Moreover, the result of this pooling of their individual abilities is that actions become possible (like the lifting of the long plank) that were not possible for each of the participants acting as individuals ^11^. 

Consider for instance a dyad using a two-handed saw to cut through a fallen tree. Person A takes control of the left side of the saw, while person B controls the right side of the saw. The affordances offered by the saw are to use a technical term, ’nested’ in the wider context of the activity of sawing the piece of wood in order to build something together. Possibilities for action are often nested within other large-scale affordances ( Van Dijk & Rietveld, 2021). The affordances of the saw in this example are embedded within the affordances of the shed where the building activity is taking place.

Dealing adequately with matters of shared relevance (like the liftability of the long plank) will require multiple individuals to combine their abilities. The result of this pooling of abilities is that affordances that are of shared relevance stand out from the landscape of affordances. Tending towards an improved grip on the situation is now something that individuals do in collaboration by acting together. They sense as a member of a dyad, or a larger group, how well or badly they are faring in their skilled engagement with the environment ^12^. In the context of the planks experiment the participants can simply take it for granted that they are doing the experiment together with another cooperative participant prepared to do what the experimenter has asked of them. However, this is not something that can ordinarily be so readily assumed. If I am to embark on some joint endeavour with another agent, I must know that they are truly committed, and they must know the same about me. It might then be argued that for both of us to know this about each other will require each of us to first identify and ascribe intentions to the other, and perhaps even to know that we have done so.

How is it that the two agents managed to act in coordinated ways if they do not represent who is doing what, when and how? We suggest that the coordination of patterns of action readiness emerges across the individuals as a dyad in just the same way as it happens within each of them based on the self-organising dynamics of the agent-agent-environment system. We have seen a simple example of this self-organising dynamic in the plank lifting experiment. The two individuals act in coordinated ways because both are responding to a situation of shared relevance (on the basis of abilities that they have acquired thanks to a history of interactions in the same form of life). It is their mutual responsiveness to the same nested structure of affordances in the shared context of the experiment that accounts for how they are able to coordinate with each other. If a couple are visiting a DIY-store together as part of a shopping trip for a home renovation, they share a readiness for finding the items they need for the renovation, engaging with the shop-assistants to help them find said items, paying for them, and returning home ^13^. Their activities are nested together in this way because the couple are each responsive to the large-scale affordance of the home renovation.

Similarly, in the plank lifting experiment, each agent is not only responsive to the affordance of liftability but to a whole nested structure situated in the living environment: to an entire field of relevant affordances. The readiness to lift the plank occurs in the wider context of the moving conveyor belt, as part of an experimental set-up in which the two subjects have been instructed. We are suggesting that each of these aspects (the plank, the moving conveyor belt, the experimental instructions) of the situation (and many others) should be thought of as providing relevant affordance that elicit a corresponding state of action readiness in each of the agents. The states of bodily action readiness are internal states of the individual that become coordinated with each other within the individual in such a way that they tend towards a grip on the nested structure of their field of relevant affordances as a whole. We can see this in the temporal dynamics of the individual’s skilled behaviour. Each affordance-related state of action-readiness operates over different time scales. For instance, some states of action-readiness relate to the possible actions invited by the linguistic instructions of the experimenters, operating over longer time scales of the duration of the whole experiment. These slower states of action-readiness enslave the faster-changing states of action readiness that relate to the planks and to their movement on the conveyor belt.

Now it is not only the states of action readiness of the individuals that are coordinated with the nested structure of affordances in the field, and to the wider sociomaterial practices they share. Insofar as the two individuals are responding to relevant affordances, they will have similar or complementary bodily states of action readiness that are coordinated with each other. Instead of moving planks now imagine that a group of people are engaged in moving a heavy piano down some stairs. The movements of each person will have to complement each other if they are to avoid losing grip on the piano. Each person will however be moving in a slightly different way, some providing more of a supporting role for the piano while other will take more of a responsibility for moving the piano downwards. It is crucial that all the individual movements that are being made are carefully and tightly adjusted to each other. The affordances of the heavy piano play a crucial role in this process, as of course do those of the stairs the people are descending. The agents are continuously adjusting their movements to each other, but only in relation to the effect their respective movements are having on the moving of the piano in the wider context of the stairs. If the moving situation becomes so precarious that they feel they need an extra pair of hands, the possibility of ringing the neighbour’s doorbell to ask them for help may become an affordance of shared relevance.

In joint action multiple individuals succeed in coordinating with each other because they form what we have called an interpersonal synergy. (We borrow this terminology from Dale *et al.*, 2014). The movement scientist Nikolai Bernstein observed that the number of muscles and joints in the human body creates a problem of control ( Bernstein, 1967; Kelso, 2009; Turvey, 1990). Each individual muscle and joint has multiple degrees of freedom. For each action the agent is ready to perform, there are very many different constellations of muscle and joint dynamics that can bring about the same action. Yet when we move our body, all of our muscles and joints work together in concert in ways that are somehow adjusted and fine-tuned to the affordances to which the agent is responding. If the individual is writing with a pencil for instance, this activity constrains the timing and force of contraction and relaxation of the individual muscles and joints in his hands, fingers, and arms. The joints and muscles appear not to act independently but interdependently, self-organising through their coupling, constraining each other’s degrees of freedom, and thereby reducing the need for control. Similarly, when several people are moving a piano, it is the constraints that derive from people being coupled to the piano that reduce the degrees of freedom of their individual movements allowing them to function together as a single coordinated whole. The mutual adaptation makes the behaviour of the individuals interdependent in a way that makes it possible for them to pool their skills, so as to coordinate their responsiveness to multiple affordances of shared relevance. It is the affordances of the heavy piano in the situation of descending the steep stairs that invites the individuals carrying the piano together to coordinate their actions in a particular way. There is no need for them to plan who does what and when. Instead, this is settled on the fly through the interpersonal synergy they form as they couple to the piano in the particular local landscape of affordances encountered on the stairs.

In the final section, we pull together the two elements we have laid out so far of a field of relevant affordances containing affordances of shared relevance and interpersonal synergies to propose an account of we-intentionality in terms of skilled intentionality. We will see that interpersonal synergies form through engagement with affordances of shared relevance. These affordances constrain and limit the degrees of freedom in the behaviour of the interlocutors. The coordination that is achieved is best accounted for in terms of the behaviour of a single integrated system that is jointly shaped by the co-actors as they respond to the affordances of shared relevance on the basis of the skills they have developed by taking part in sociomaterial practices.

## Skilled we-intentionality

In the previous section, we saw how it is the affordances of the joint liftability of the piano nested in the setting of the stairs around which the interpersonal synergy self-organises. The multiple nested affordances that make up the situation in which the piano is being moved together function as constraints on the self-organising process. Each relevant affordance generates micro-states of action readiness. Multiple simultaneous states of action readiness self-organise to form a macro-pattern of action readiness across the two individuals (an interpersonal synergy) in ways that are constrained by affordances of shared relevance. The affordances of the piano moving situation provide constraints on the behaviour of the individuals. Their skills attune them to this dynamically changing and evolving situation as they move the piano allowing them to maintain a good grip on the piano.

A central question for a theory of joint action, seen from this ecological dynamical perspective, is under what conditions affordances stand out as being of shared relevance inviting the coordinated actions of multiple individuals. We’ve seen (in section two with the example of the football player) how affordances stand out as inviting to an individual when they improve grip. One is ready to act on those relevant affordances that allow one to tend towards a better grip. The same is true when affordances are of shared relevance. Affordances stand out as of shared relevance when it is only by acting in ways that are mutually adapted that each of the individuals is able to improve grip. Suppose one is using a two-handed saw. This is the kind of saw that you can only use to cut through a fallen tree if when one agent pushes on the saw, the other agent pulls. The rhythm the two agents establish is thus crucial to using the saw skilfully. The two agents need to mutually adapt to each other producing complementary actions, just like in the case of the piano we just discussed. If they do not adapt to each other, they will have the sense as a dyad that what they are doing is not going well. So long as they mutually adapt each allowing their actions to be drawn from them by the affordances of the two-handed saw, they will succeed in improving grip as a dyad. Thus, affordances will be experienced as shared invitations to multiple individuals when each of the individuals has the sense that a way to improve grip is by joining forces with other agents. We can suppose that this is a sense the individuals develop often through interaction with others. We learn that there are certain affordances such as those of two-handed saws that can only be engaged with skilfully by doing things together with other people.

We-intentionality, which is typically taken to be required for acting jointly, can therefore be explained in terms of affordances of shared relevance that act as constraints on the self-organising states of action readiness. The agents that participate in a joint action do not need to have predefined intentions or goals that specify who is to do what and when, in order to coordinate with each other. Multiple agents can have control over an action the states of action readiness self-organise to form interpersonal synergies in response to affordances of shared relevance.

It might be objected that people often do act based on explicit goals and intentions that they set for themselves in advance of acting ^14^. The rich literature on planning to which Michael Bratman for instance has made important contributions is all about the cognitive dynamics of goal-setting ( Bratman, 1987; cf. Castelfranchi & Paglieri, 2007; Pacherie, 2006). We have shown on the basis of ethnographic research over the course of many months in the practice of making art & architecture that even cases of long-term planning can naturally be described in terms of skilled intentionality ( Rietveld *et al.*, 2022; van Dijk & Rietveld, 2017; van Dijk & Rietveld, 2020; van Dijk & Rietveld, 2021). The essence of what we found is that skilled agents, in this case visual artists and architects, are able to be responsive to large scale affordances, like the possibility to create an art installation that had never been made before and took five years to realize.

A simple example on a much shorter timescale might help to drive this point home. Suppose that I have agreed with my partner that I will cook tonight, and I am thinking about the ingredients I will need. The dish is one I’ve made many times before and because of my extensive practice I know exactly what to do to make it taste its best. I know in particular which vegetables and spices are needed. Yet, I don’t have what I need and this situation makes the visit to the supermarket an attractive course of action. A supermarket visit is a possibility for action (a large-scale affordance) available in our human form of life and one with which I am very familiar. All of the thinking and planning in this example, can be readily made sense of in terms of my skilled engagement with this large-scale affordance over the time-scale of a few hours ( Van Dijk & Rietveld, 2021). We would therefore resist making a distinction between emergent coordination and planned coordination as Knoblich *et al.* 2011 propose to do. We propose to understand all cases of coordination of action by multiple individuals as self-organized in terms of skilled intentionality. Examples of future-directed actions that seem to call for explanation in terms of who will do what and when, we propose to explain in terms of responsiveness to large-scale affordances such as visits to the supermarket that serve to organise the relevant affordances whose invitations weave together over time. Understanding action in terms of skilled intentionality allows for an explanation of how the individual is always ready for multiple possibilities that matter to them even when some of these possibilities are some way off in the future.

Consider, as a final example ostensive communication through pointing. Suppose we are out collecting fruit together and I point at some berries but all you can see are leaves. You can readily infer that I am not pointing at the leaves but most likely at some hidden berries. One might suppose that you can make this inference because the shared goal of finding berries is part of our common ground ( Tomasello, 2014). There are many possible features of the current perceptual scene I might be attempting to direct your attention towards. To identify which of these features is actually of relevance to you, you must first determine what it is that I am intending to make you believe. You must think something along the following lines: his pointing in the direction of those leaves would make sense if he intends to make me believe there are berries hidden over there behind those leaves. Similarly, when I make the pointing gesture, I am also imagining how the gesture is likely to be received by you, and whether it is likely to be understood. I must think about what my partner will infer about my intentions, and the beliefs I intend to bring about in her. In contrast to this, we’ve argued that in perceiving the pointing gesture, you will be able to immediately recognise that I mean to draw your attention to the hidden berries, not to the leaves. It is the hidden berries that are of shared relevance to us in this situation. We are after all, engaged in the shared activity of hunting for berries. The pointing gesture is made in a situation that is of shared relevance because we are undertaking this activity together.

## Conclusion

Our aim in this paper has been to situate we-intentionality in relation to the social, cultural, material and natural environment, what we have called the ‘living environment’. We have shown how people can succeed in acting together in coordinated ways without reasoning about each other’s intentions. We have proposed an account of we-intentionality in terms of skilled intentionality, the coordination with multiple affordances simultaneously. When people coordinate with each other, they are responsive to (multiple) affordances of shared relevance. We have explained this responsiveness in terms of multiple states of bodily action readiness that self-organise in ways that are enabled and constrained by the affordances of shared relevance. Typically, people are able to coordinate with each other on the basis of skills for responding to affordances of shared relevance.

We’ve asked the concept of shared relevance to do a good deal of explanatory work in the account of joint action we have proposed. The key question in the account of joint action we propose is why it should be that an affordance (like the liftability of the plank) should stand out from the landscape of affordances as inviting or calling for the coordinated action of multiple individuals. Why should it be that individuals sometimes find themselves ready to join forces and collaborate in responding to affordances of shared relevance? The answer to this question is just the same as the answer we would give in the case of skilled intentionality for the actions of individuals. Affordances stand out as relevant for an individual agent because of disattunement in the agent-environment system as a whole, which the individual then acts to reduce. Affordances are of shared relevance, we have argued, when the only way to improve grip is by forming an interpersonal synergy with other individuals, like in the example of the two-handed saw. Affordances more generally stand out from the landscape as being of shared relevance to two or more individuals because of the sociomaterial practice of which the individuals are members. The berries stand out as relevant for the people that are foraging together for instance, because foraging is part of their shared way of life, and the berries are significant in the context of this shared activity. 

Now it might be objected that all skilled intentionality turns out to be skilled-we intentionality. For whenever people act skilfully they are coordinating with other members of a practice. What this objection highlights is that there is indeed something common to the skilled actions of individuals, dyads and groups. What is common are the multiple relevant affordances and the shared sociomaterial practices with which individuals are coordinating when they act with skill. However, we have argued that there are also differences between what individuals do when they feel, think and act as dyads, or as members of larger groups. In the latter cases, the individuals pool their sensitivities to situated normativity, their abilities for distinguishing between better or worse ways of acting, so as to tend towards an improved grip on affordances of shared relevance as members of a dyad or group. The individuals are ready to join forces with relevant affordances and act together based on what matters to them as members of a practice.

## Data availability

No data are associated with this article.

## References

[ref-1] Abramova K Slors M : Social cognition in simple action coordination: a case for direct perception. *Conscious Cogn.* 2015;36:519–531. 10.1016/j.concog.2015.04.013 26003382

[ref-2] Beer RD : Dynamical approaches to cognitive science. *Trends Cogn Sci.* 2000;4(3):91–9. 10.1016/s1364-6613(99)01440-0 10689343

[ref-3] Bernstein NA : Coordination and Regulation of Movement.New York: Pergamon Press, 1967. Reference Source

[ref-4] Bratman ME : Shared Agency: a Planning Theory of Acting Together.Oxford: Oxford University Press, 2014. Reference Source

[ref-6] Bratman ME : Intentions, Plans and Practical Reason.Cambridge, MA: Harvard University Press,1987. Reference Source

[ref-5] Bratman ME : Shared intention. *Ethics.* 1993;104(1):97–113. Reference Source

[ref-8] Bruineberg J Rietveld E : Self-organization, free energy minimization, and optimal grip on a field of affordances. *Front Hum Neurosci.* 2014;8:599. 10.3389/fnhum.2014.00599 25161615 PMC4130179

[ref-7] Bruineberg J Rietveld E : What’s inside your head once you’ve figured out what your head is inside of. *Ecol Psychol.* 2019;31(3):198–217. 10.1080/10407413.2019.1615204

[ref-9] Bruner J : Child’s Talk.New York, NY: Norton, 1983.

[ref-10] Butterfill SA : Joint action and development. *Philos Q.* 2012;62(246):23–47. 10.1111/j.1467-9213.2011.00005.x

[ref-11] Castelfranchi C Paglieri F : The role of beliefs in goal dynamics: prolegomena to a constructive theory of intentions. *Synthese.* 2007;155(2):237–263. Reference Source

[ref-12] Chemero A : Sensorimotor empathy. *J Conscious Stud.* 2015;23(5–6):138–153. Reference Source

[ref-13] Chemero A : Radical Embodied Cognitive Science.Cambridge, MA: MIT Press, 2009. Reference Source

[ref-120] Dewey J : Art as Experience.New York: Minton Balch.1934. Reference Source

[ref-90] Dale R Fusaroli R Duran ND : The self-organization of human interaction.In B. Ross (Ed.) *The Psychology of Learning and Motivation.*Burlington MA,: Elsevier Academic Press,2014;54:43–95. 10.1016/B978-0-12-407187-2.00002-2

[ref-15] Dreyfus HL : Why Heideggerian AI failed and how fixing it would require making it more Heideggerian. *Philos Psychol.* 2007;20(2):247–68. 10.1080/09515080701239510

[ref-16] Freeman WJ : How Brains Make Up Their Minds.New York: Columbia University Press, 2000. Reference Source

[ref-77] Freeman WJ : Simulation of chaotic EEG patterns with a dynamic model of the olfactory system. *Biol Cybern.* 1987;56(2–3):139–50. 10.1007/BF00317988 3593783

[ref-17] Frijda NH : The emotions.New York: Cambridge University Press, 1986. Reference Source

[ref-18] Frijda NH : The Laws of Emotion.Mahwah, NJ: Lawrence Erlbaum Associates, Inc. 2007. Reference Source

[ref-75] Friston K : The free energy principle: a unified brain theory? *Nat Rev Neurosci.* 2010;11(2):127–38. 10.1038/nrn2787 20068583

[ref-91] Friston K : Embodied Inference: or “I think therefore I am, if I am what I think”. *The implications of embodiment (Cognition and Communication).* 2011;89–125. Reference Source

[ref-76] Fusaroli R Tylén K : Carving language for social interaction: a dynamical approach. *Interaction Studies.* 2012;13(1):103–24. 10.1075/is.13.1.07fus

[ref-20] Gallagher S Ransom T : Artifacting minds: material engagement theory and joint action.In C Tewes (Ed.) *Embodiment in Evolution and Culture.*Berlin: De Gruyter,2016;337–51. Reference Source

[ref-21] Gibson JJ : The Ecological Approach to Visual Perception.New York: Psychology Press;1979/2014. Reference Source

[ref-23] Gilbert MP : On Social Facts.Princeton, NJ: Princeton University Press, 1989. 10.2307/j.ctv10vm20z

[ref-22] Gilbert MP : Living Together: Rationality, Sociality, and Obligation.London: Rowman & Littlefield. 1996. Reference Source

[ref-92] Gilbert M : Joint Commitment: How We Make the Social World.Oxford, UK: Oxford University Press.2014. 10.1093/acprof:oso/9780199970148.001.0001

[ref-24] Haugeland J : The intentionality all-stars. *Philos Perspect.* 1990;4:383–427. 10.2307/2214199

[ref-26] Heft H : Ecological Psychology in Context: James Gibson, Roger Barker, and the Legacy of William James’ Radical Empiricism. New York: Taylor & Francis/Psychology Press,2001. 10.4324/9781410600479

[ref-25] Hobson P : The Cradle of Thought.Reading, UK: Palgrave Macmillan, 2002. Reference Source

[ref-27] Käufer S Chemero A : Phenomenology – An Introduction. Cambridge, UK: Polity Press,2015. 10.19079/pr.2015.11.tan

[ref-29] Kelso JAS : Dynamic Patterns: The Self-Organisation of Brain and Behaviour. Cambridge, MA: MIT Press,1995. Reference Source

[ref-28] Kelso JAS : Synergies: atoms of brain and behavior. *Adv Exp Med Biol.* 2009;629:83–91. 10.1007/978-0-387-77064-2_5 19227496

[ref-30] Kiverstein J Rietveld E : Scaling-up skilled intentionality to linguistic thought. *Synthese.* 2021;198:175–194. Special Issue – Radical Theories of Cognition. 10.1007/s11229-020-02540-3

[ref-31] Kiverstein J van Dijk L Rietveld E : The field and landscape of affordances: Koffka’s two environments revisited. *Synthese: Special Issue Gestalt Psychology, Phenomenology and Embodied Cognitive Science.* 2019. 10.1007/s11229-019-02123-x

[ref-32] Knoblich G Butterfill S Sebanz N : Psychological research on joint action: theory and data. In B Ross (Ed.) *The Psychology of Learning and Motivation.*Burlington MA,: Elsevier Academic Press,2011;54:59–101. 10.1016/B978-0-12-385527-5.00003-6

[ref-33] Ladyman J Wiesner K : What is a Complex System?London, UK: Yale University Press,2020. 10.2307/j.ctv14rmpwc

[ref-93] List C Pettit P : Group Agency: The Possibility, Design, and Status of Corporate Agents.Oxford, UK: Oxford University Press.2011. 10.1093/acprof:oso/9780199591565.001.0001

[ref-34] Malafouris L : How Things Shape the Mind: A Theory of Material Engagement. Cambridge, MA: MIT Press,2013. Reference Source

[ref-35] Marsh KL : Social ecological context of conversing. *Ecol Psychol.* 2015;27(4):310–334. 10.1080/10407413.2015.1086229

[ref-37] Merleau-Ponty M : The Structure of Behaviour. Translated by Alden L Fisher, Boston: Beacon Press,1943/1962.

[ref-36] Merleau-Ponty M : Phenomenology of Perception. Translated by Colin Smith, London: Routledge,1945/2002. 10.4324/9780203994610

[ref-38] Mol A : The Body Multiple: Ontology in Medical Practice. Durham, NC: Duke University Press,2002. 10.2307/j.ctv1220nc1

[ref-94] Noë A : Strange Tools: Art and Human Nature.New York, NY: Hill & Wang.2015. 10.3202/caa.reviews.2018.90

[ref-39] Orlikowski WJ : Sociomaterial practices: Exploring technology at work. *Organization Studies.* 2007;28(9):1435–1448. 10.1177/0170840607081138

[ref-41] Pacherie E : Toward a dynamic theory of intentions. In S Pockett, WP Banks, and S Gallagher (Ed’s) *Does Consciousness Cause Behaviour? An Investigation of the Nature of Volition.*(Cambridge MA: MIT Press),2006;145–167. 10.7551/mitpress/9780262162371.003.0009

[ref-40] Pacherie E : Is collective intentionality really primitive. M Beaney, C Penco, & M Vignolo (Ed.’s) *Mental Processes: Representing and Inferring.*Cambridge, UK: Cambridge Scholar’s Press,2007;153–175. Reference Source

[ref-95] Rakoczy H : The development of individual and shared intentionality.In J Kiverstein (Ed.) *The Routledge Handbook of Philosophy of the Social Mind.*London: Routledge,2017;139–151.

[ref-42] Reddy V : Why engagement?: A second-person take on social cognition. In A Newen, L De Bruin & S Gallagher (Ed’s) *The Oxford Handbook of 4 ^E^ Cognition.*Oxford, UK: Oxford University Press,2018;433–453. 10.1093/oxfordhb/9780198735410.013.23

[ref-44] Reed ES : Encountering the world: Toward an ecological psychology. New York, NY: Oxford University Press,1996. Reference Source

[ref-43] Reed ES Bril B : The primacy of action in development: A commentary on N. Bernstein. *Dexterity and its Development.*(Translated, by ML Latash, Edited by ML Latash, MT Turvey), Mahwah NJ: Lawrence Erlbaum Associates Publishers,1996;431–451. Reference Source

[ref-45] Reed ES : Cognition as the cooperative appropriation of affordances. *Ecol Psychol.* 1991;3(2):135–158. 10.1207/s15326969eco0302_5

[ref-78] Richardson MJ Campbell WL Schmidt RC : Movement interference during action observation as emergent coordination. *Neurosci Lett.* 2009;449(2):117–122. 10.1016/j.neulet.2008.10.092 18996439

[ref-46] Richardson MJ Marsh KL Schmidt RC : Challenging the egocentric view of coordinated perceiving, acting and knowing. In LF Barrett, B Mesquita, & ER Smith (Ed’s) *Mind in Context.*New York: Guilford Press,2010;307–33. Reference Source

[ref-47] Richardson MJ Marsh KL Baron RM : Judging and actualizing intrapersonal and interpersonal affordances. *J Exp Psychol Hum Percept Perform.* 2007;33(4):845–859. 10.1037/0096-1523.33.4.845 17683232

[ref-97] Rietveld E : Situated Normativity: The Normative Aspect of Embodied Cognition in Unreflective Action. *Mind.* 2008;117(468):973–1001. 10.1093/mind/fzn050

[ref-48] Rietveld E Denys D van Westen M : Ecological-enactive cognition as engaging with a field of relevant affordances: The skilled intentionality framework (SIF). In A Newen, L De Bruin, & S Gallagher (Eds.), *The Oxford handbook of 4E (embodied, embedded, extended, enactive) cognition.*Oxford: Oxford University Press,2018;41–70. 10.1093/oxfordhb/9780198735410.013.3

[ref-49] Rietveld E Kiverstein J : A rich landscape of affordances. *Ecol Psychol.* 2014;26(4):325–352. 10.1080/10407413.2014.958035

[ref-96] Rietveld E Rietveld R Martens J : Collaborative embodied engagements with strategic interventions: a conversation.In K. Bicknell & J. Sutton (Ed’s) *Collaborative Embodied Performance: Ecologies of Skill.*London, UK: Methuen Drama, Bloomsbury Publishing.2022.

[ref-98] Rochat P Robbins E : Sharing and fairness in development.In J. Kiverstein (Ed.) *The Routledge Handbook of Philosophy of the Social Mind.*London: Routledge,2017;222–243. 10.4324/9781315530178.ch13

[ref-50] Rogoff B : Apprenticeship in Thinking: Cognitive Development in Social Context. Oxford, UK: Oxford University Press,1990. Reference Source

[ref-51] Satne G : Interaction and self-correction. * Front Psychol.* 2014;5:798. 10.3389/fpsyg.2014.00798 25101044 PMC4107969

[ref-99] Satne G Sallice A : Shared intentionality and the cooperative evolutionary hypothesis.En. A. Fiebich (Ed.). *Minimal Cooperation and Shared Agency.* New York, NY: Springer,2020;71–92. 10.1007/978-3-030-29783-1_5

[ref-53] Searle JR : Collective intentions and actions. In P. Cohen, J. Morgan & M.E. Pollack (Ed’s) *Intentions in Communication.* Cambridge: Cambridge University Press,1990;90–105. Reference Source

[ref-52] Searle JR : The Social Construction of Reality. New York: Free Press.1995. Reference Source

[ref-100] Segundo-Ortin M Satne G : Sharing attention, sharing affordances: from dyadic interaction to collective information.In D. D’Angelo, M. Merle & E. Solomonova (Ed’s) *Mediation and Access. A New Approach to Attention*. De Gruyter: Berlin. (forthcoming). Reference Source

[ref-54] Sellars W : Science, Perception and Reality. London: Routledge & Kegan Paul.1963. Reference Source

[ref-55] Shockley K Richardson DC Dale R : Conversation and coordinative structure. * Top Cogn Sci.* 2009;1(2):305–319. 10.1111/j.1756-8765.2009.01021.x 25164935

[ref-56] Sterelny K : The Evolved Apprentice.Cambridge, MA: MIT Press.2012. Reference Source

[ref-57] Strogatz SH : Non-Linear Dynamics and Chaos: With Applications to Physics, Biology, Chemistry and Engineering. New York, NY: Perseus Books.1994. Reference Source

[ref-58] Suchman LA : Plans and Situated Actions: the Problem of Human Machine Interaction. Cambridge: Cambridge University Press.1987. Reference Source

[ref-59] Tollefsen D Dale R : Naturalising joint action: a process-based approach. *Philos Psychol.* 2012;25(3):385–407. 10.1080/09515089.2011.579418

[ref-60] Tomasello M : A Natural History of Human Thinking. Cambridge, MA: Harvard University Press.2014. Reference Source

[ref-61] Tschacher W Haken H : Intentionality in non-equilibrium systems? The functional aspects of self-organised pattern formation. *New Ideas Psychol.* 2007;25(1):1–15. 10.1016/j.newideapsych.2006.09.002

[ref-62] Tsuda I : Towards an interpretation of dynamic neural activity in terms of chaotic dynamical systems. * Behav Brain Sci.* 2001;24(5):793–809. 10.1017/s0140525x01000097 12239890

[ref-63] Tuomela R : We-intentions revisited. *Philosophical Studies.* 2005;125(3):327–369. 10.1007/s11098-005-7781-1

[ref-64] Tuomela R Miller K : We-intentions. *Philosophical Studies.* 1988;53:367–389. 10.1007/BF00353512

[ref-65] Turvey MT : Coordination. *The American Psychologist.* 1990;45:938–953. 10.1037/0003-066X.45.8.938 2221565

[ref-121] Van Den Herik J Rietveld E : Reflective situated normativity. *Philosophical Studies.* 2021. 10.1007/s11098-021-01605-4

[ref-66] Van Dijk L Rietveld E : Situated imagination. *Phenomenology & Cognitive Sciences.* 2020. 10.1007/s11097-020-09701-2 PMC11910435 40099166

[ref-67] Van Dijk L Rietveld E : Situated anticipation. *Synthese.* 2021;198(1):349–371. 10.1007/s11229-018-02013-8 33583961 PMC7822785

[ref-68] Van Dijk L Rietveld E : Foregrounding sociomaterial practice in our understanding of affordances: the skilled intentionality framework. *Front Psychol.* 2017;7:1969. 10.3389/fpsyg.2016.01969 28119638 PMC5220071

[ref-101] Vincini S Gallagher S : Developmental phenomenology: examples from social cognition. *Cont Philos Rev.* 2021;54(2):183–199. 10.1007/s11007-020-09510-z

[ref-69] Vygotsky LS : Mind in Society: The Development of Higher Psychological Processes.(Ed.’s M Cole, VJ Steiner, S Scribner, & E Souberman), Cambridge, MA: Harvard University Press.1978. 10.2307/j.ctvjf9vz4

[ref-70] Withagen R de Poel HJ Araujo D : Affordances can invite behavior: Reconsidering the relationship between affordances and agency. *New Ideas Psychol.* 2012;30(2):250–258. 10.1016/j.newideapsych.2011.12.003

