# Supplementary material for: Skilled we-intentionality: Situating joint action in the living environment
Source: Open Res Eur. 2021 Sep 27;1:54. Originally published 2021 May 21. [Version 2] doi: 10.12688/openreseurope.13411.2 (PMC10445857; doi:10.12688/openreseurope.13411.2)
Supplement: Peer review history

# Peer review: Reviewer response for version 2

**DOI:** 10.21956/openreseurope.15233.r27687

**Published:** 2021-10-15

**Authors:** Glenda Satne

**Affiliations:** 1 Philosophy, School of Humanities and Social Inquiry, Faculty of Arts, Social Sciences and Humanities, University of Wollongong, Wollongong, Australia

I have reviewed the authors replies and the updated paper, I am happy to accept the paper as it is, without further revisions.

If any, are all the source data and materials underlying the results available?

No source data required

Is the work original in terms of material and argument?

Yes

Is the work clearly and cogently presented?

Partly

Is the argument persuasive and supported by evidence?

Partly

Does the research article contribute to the cultural, historical, social understanding of the field?

Yes

Does it sufficiently engage with relevant methodologies and secondary literature on the topic?

Yes

Reviewer Expertise:

NA

I confirm that I have read this submission and believe that I have an appropriate level of expertise to confirm that it is of an acceptable scientific standard.

# Peer review: Reviewer response for version 2

**DOI:** 10.21956/openreseurope.15233.r27686

**Published:** 2021-10-05

**Authors:** Anthony Chemero

**Affiliations:** 1 Departments of Philosophy and Psychology, University of Cincinnati, Cincinnati, OH, USA

The authors have addressed my concerns. I approve this paper for indexing.

If any, are all the source data and materials underlying the results available?

No source data required

Is the work original in terms of material and argument?

Yes

Is the work clearly and cogently presented?

Yes

Is the argument persuasive and supported by evidence?

Yes

Does the research article contribute to the cultural, historical, social understanding of the field?

Yes

Does it sufficiently engage with relevant methodologies and secondary literature on the topic?

Yes

Reviewer Expertise:

NA

I confirm that I have read this submission and believe that I have an appropriate level of expertise to confirm that it is of an acceptable scientific standard.

# Peer review: Reviewer response for version 1

**DOI:** 10.21956/openreseurope.14481.r26951

**Published:** 2021-07-22

**Authors:** Anthony Chemero

**Affiliations:** 1 Departments of Philosophy and Psychology, University of Cincinnati, Cincinnati, OH, USA

Comments on Kiverstein and Rietveld, “Skilled We-intentionality”, 2021

 This paper is a valuable notification for philosophers who work on social and group cognition that ecological and dynamical psychologists have been building up a substantial body of work that is directly relevant to their concerns. These psychologists have built up an expanding body of empirical results and theoretical arguments that explain joint action without any reference to mental representations—whether individual, joint, or aligned. Kiverstein and Rietveld argue that shared affordances structure joint actions, without representations. This is in direct contradiction to the assumptions that have structured the philosophical debate. Because Kiverstein and Rietveld cite all the key players in the philosophical debate, those key players are much more likely to pay some attention than they have. Most of us in the ecological and dynamical communities typically ignore these philosophers; and they ignore us right back. (I have been guilty of this in my own writing on joint action.) This paper is a worthwhile contribution and worth publishing for that reason alone.

 That said, I do have one major concern about the role of the Skilled Intentionality Framework in the argument. Let me preface the concern by saying that I am a fan of the Skilled Intentionality Framework and am even an author on one of the papers promoting and elaborating it. However, some of my colleagues in the ecological and dynamical psychology community complain that the Skilled Intentionality Framework is primarily a re-branding of ideas that were already in the scientific literature, and that its only innovations are terminological (and so unnecessary). I do not share this complaint, and defend the Skilled Intentionality Framework  when it is raised. But it does align with my main concern with this paper. How much argumentative work is the Skilled Intentionality Framework actually doing in this paper?

 The main argumentative structure of the paper goes from affordances, via development, to shared affordances to social coordination dynamics to interpersonal synergies to self-organized joint action on shared affordances, which obviates the need for mental representations in the explanation of joint action. This is a compelling argument. (In fact, I am finishing a book that follows some of the same path.) But notice that I did not mention the Skilled Intentionality Framework. What does the Skilled Intentionality Framework add to the mix? More importantly, what does the Skilled Intentionality Framework add that isn’t already there from ecological psychologists like Reed and Heft and Turvey and Shaw?

 In my opinion, the main contribution of the Skilled Intentionality Framework here is the distinction affordances and invitations, but that distinction doesn’t get much discussion. Affordances are opportunities for action; invitations are those opportunities for action that align with current goals and motivations. Kiverstein and Rietveld draw on Merleau-Ponty’s idea of motor intentionality, which they call ‘skilled intentionality’ to bridge this gap between what we can do and what we might actually do. A second smaller contribution of the Skilled Intentionality Framework is the story it tells about the shared maintenance of cultural affordances. The latter of these is already available in the work of Reed and Heft that they cite. The former might be available in Shaw’s work on intentional dynamics which they don’t cite (and which I am not sure I understand).  This leads to the worry that the primary contribution of the Skilled Intentionality Framework really is terminological in this case. What Kiverstein and Rietveld have done, according to this worry, is used some new terminology (‘skilled intentionality’, ‘the landscape of affordances’, ‘the field of affordances’) to set out some old ideas.

 As I said above, this paper is worthwhile as is despite this concern just for the likelihood that it will lead to some cross-pollination between philosophers of joint action and ecological psychologists. But I do think that Kiverstein and Rietveld should make much more clear exactly what the Skilled Intentionality Framework is adding to the argument, and differentiating between that and what is already available in the ecological and dynamical psychology literatures. This is important not just for this paper, but for the Skilled Intentionality Framework more generally.

If any, are all the source data and materials underlying the results available?

No source data required

Is the work original in terms of material and argument?

Yes

Is the work clearly and cogently presented?

Yes

Is the argument persuasive and supported by evidence?

Yes

Does the research article contribute to the cultural, historical, social understanding of the field?

Yes

Does it sufficiently engage with relevant methodologies and secondary literature on the topic?

Yes

Reviewer Expertise:

philosophy of cognitive science, ecological psychology, dynamic systems theory

I confirm that I have read this submission and believe that I have an appropriate level of expertise to confirm that it is of an acceptable scientific standard, however I have significant reservations, as outlined above.

# Response

**Published:** 2021-09-08

**Authors:** Julian Kiverstein

**Affiliations:** Amsterdam University Medical Research, Netherlands Antilles

**Clarifying our aims**

 The aim of this paper was to connect research on social coordination in ecological psychology with our theoretical research on affordances, along the same lines as an earlier publication (Van Dijk & Rietveld 2017). We have argued in previous work for a theory that defines the affordances of the human environment in relation to a shared social, cultural and material form of life. This work has been inspired by among others Harry Heft and Ed Reed (Heft 2001; Reed 1996). Some of the research on social coordination that has been done in ecological psychology (e.g. Richardson *et al*. 2007) has been taken up in the philosophical literature on joint action by philosophers like Stephen Butterfill (e.g. Knoblich *et al *2011) and Deborah Tollefsen (e.g. Tollefsen & Dale 2011). However, these philosophers have also tended to insist on a distinction between a form of social coordination which is emergent, and well-described by ecological psychology, and a form of joint action that is planned and calls for a more traditional cognitive explanation in terms of shared intentions and goals. More specifically, we tried to bring our research on affordances to bear to show that such a distinction between planned and emergent coordination is not needed. This led us to think about how to generalise our work on skilled intentionality, which has up until now focused on how the skilled individual is ready to respond to multiple relevant affordances at the same time, to thinking about skilled actions performed by dyads or groups. We wanted to show how the concept of skilled intentionality can be applied to a domain of action typically thought to call for higher-cognition, namely we-intentionality. Indeed, we-intentionality is sometimes argued to be associated with distinctively human forms of thinking and acting – see, for example the work of Michael Tomasello (2019). Our aim was to show how SIF might be able to make sense of what is according to some a uniquely human form of thinking and acting. If such a project succeeds it would correct for an often heard complaint that research in ecological-enactive cognitive science while well-suited for understanding perception and action will not scale up to explain what is distinctive about human cognition (see also Rietveld & Kiverstein 2014; Kiverstein & Rietveld 2018, 2020).

# Response

**Published:** 2021-09-08

**Authors:** Julian Kiverstein

**Affiliations:** Amsterdam University Medical Research, Netherlands Antilles

2. **Is SIF largely "an exercise in rebranding"?**

 One of the core concepts of our paper – interpersonal synergies – is indeed borrowed from the literature in ecological psychology on dynamics of social interaction in conversation (Dale *et al*. 2014). The novel contribution of our paper is however to describe how interpersonal synergies self-organise in relation to the field of relevant affordances. This is not an idea that is present in the earlier literature but it is one that opens up to us because of the distinction we make between affordances and invitations based on the phenomenology of skilled action, and between the landscape of available affordances and the field of relevant affordances based on our own earlier work on the philosophy of affordances. Of course, both the concept of affordances and that of invitations were also present in the phenomenological literature prior to our developing SIF. The distinction between the landscape of affordances and the field of relevant affordances was introduced by us. As we explained at the beginning of our reply, we developed SIF to provide bridging concepts that could connect the phenomenological literature to work in ecological dynamics and neurodynamics.

# Response

**Published:** 2021-09-08

**Authors:** Julian Kiverstein

**Affiliations:** Amsterdam University Medical Research, Netherlands Antilles

**Large-scale affordances and temporalising affordances:**

 Finally, our recent work on large-scale affordances, and the temporalizing of the landscape of affordances, is an innovation of SIF made possible by our definition of affordances in relation to sociomaterial practices. The idea of skilled agents engaging with large-scale affordances is important in this article for resisting the distinction that Butterfill and colleagues have made between ‘emergent” and “planned” coordination. In her review of this paper, Glenda Satne suggested we should restrict the ambitions of our paper to emergent coordination. She objected that to attempt to generalise the concept of skilled intentionality to planned coordination was stretching this concept beyond its usefulness. However, we have made explicit in our reply to her above why we disagree and prefer to remain more ambitious. The landscape of affordances in the human form of life includes possibilities for actions that traditionally would have been characterized as forms of ‘higher’ cognition. On the basis of ethnographic work we have shown that this richness of the landscape allows for an account to be given of complex team activities such as creating jointly a building or artwork that does not yet exist and situated talking in terms of skilled intentionality (van Dijk & Rietveld, 2018, 2020; van den Herik & Rietveld 2020).Understanding skilled action in terms of responsiveness to multiple relevant affordances over time, including large-scale affordances, allows us to account for both emergent and planned coordination. These are important theoretical contributions of SIF that should help with the scaling-up objection often levelled against work in ecological dynamics and embodied cognition.

# Response

**Published:** 2021-09-08

**Authors:** Julian Kiverstein

**Affiliations:** Amsterdam University Medical Research, Netherlands Antilles

The main aim of the Skilled Intentionality Framework (SIF) is to provide an integrative conceptual framework allowing for connections to be made between otherwise disparate research fields of philosophical phenomenology, ecological dynamics, enaction, neurodynamics, and anthropology. The aim of this paper was to connect research on social coordination in ecological psychology with our theoretical research on affordances, along the same lines as an earlier publication (Van Dijk & Rietveld 2017). We have argued in previous work for a theory that defines the affordances of the human environment in relation to a shared social, cultural and material form of life. This work has been inspired by among others Harry Heft and Ed Reed (Heft 2001; Reed 1996). Some of the research on social coordination that has been done in ecological psychology (e.g. Richardson et al. 2007) has been taken up in the philosophical literature on joint action by philosophers like Stephen Butterfill (e.g. Knoblich et al 2011) and Deborah Tollefsen (e.g. Tollefsen & Dale 2011). However, these philosophers have also tended to insist on a distinction between a form of social coordination which is emergent, and well-described by ecological psychology, and a form of joint action that is planned and calls for a more traditional cognitive explanation in terms of shared intentions and goals. More specifically, we tried to bring our research on affordances to bear to show that such a distinction between planned and emergent coordination is not needed. This led us to think about how to generalise our work on skilled intentionality, which has up until now focused on how the skilled individual is ready to respond to multiple relevant affordances at the same time, to thinking about skilled actions performed by dyads or groups. We wanted to show how the concept of skilled intentionality can be applied to a domain of action typically thought to call for higher-cognition, namely we-intentionality. Indeed, we-intentionality is sometimes argued to be associated with distinctively human forms of thinking and acting – see, for example the work of Michael Tomasello (2019). Our aim was to show how SIF might be able to make sense of what is according to some a uniquely human form of thinking and acting. If such a project succeeds it would correct for an often heard complaint that research in ecological-enactive cognitive science while well-suited for understanding perception and action will not scale up to explain what is distinctive about human cognition (see also Rietveld & Kiverstein 2014; Kiverstein & Rietveld 2018, 2020).

 So, is this an exercise in “rebranding” ideas that are already in the scientific literature in the terms of SIF? One of the core concepts of our paper – interpersonal synergies – is indeed borrowed from the literature in ecological psychology on dynamics of social interaction in conversation (Dale et al. 2014). The novel contribution of our paper is however to describe how interpersonal synergies self-organise in relation to the field of relevant affordances. This is not an idea that is present in the earlier literature, but it is one that opens up to us because of the distinction we make between affordances and invitations based on the phenomenology of skilled action, and between the landscape of available affordances and the field of relevant affordances based on our own earlier work on the philosophy of affordances. Of course, both the concept of affordances and that of invitations were also present in the phenomenological literature prior to our developing SIF. The distinction between the landscape of affordances and the field of relevant affordances was introduced by us. As we explained at the beginning of our reply, we developed SIF to provide bridging concepts that could connect the phenomenological literature to work in ecological dynamics and neurodynamics. Finally, our recent work on large-scale affordances, and the temporalizing of the landscape of affordances, is an innovation of SIF made possible by our definition of affordances in relation to sociomaterial practices. The idea of skilled agents engaging with large-scale affordances is important in this article for resisting the distinction that Butterfill and colleagues have made between ‘emergent” and “planned” coordination. In her review of this paper, Glenda Satne suggested we should restrict the ambitions of our paper to emergent coordination. She objected that to attempt to generalise the concept of skilled intentionality to planned coordination was stretching this concept beyond its usefulness. However, we have made explicit in our reply to her above why we disagree and prefer to remain more ambitious. The landscape of affordances in the human form of life includes possibilities for actions that traditionally would have been characterized as forms of ‘higher’ cognition. On the basis of ethnographic work, we have shown that this richness of the landscape allows for an account to be given of complex team activities such as creating jointly a building or artwork that does not yet exist and situated talking in terms of skilled intentionality (van Dijk & Rietveld, 2018, 2020; van den Herik & Rietveld 2020). Understanding skilled action in terms of responsiveness to multiple relevant affordances over time, including large-scale affordances, allows us to account for both emergent and planned coordination. These are important theoretical contributions of SIF that should help with the scaling-up objection often levelled against work in ecological dynamics and embodied cognition.

     

 References Bratman, M.E. 1993. Shared intention. Ethics 104: 97-113. Bruineberg, J. & Rietveld, E. 2019. What’s inside your head once you’ve figured out what your head is inside of. Ecological Psychology 31 (3): 198-217.  Frijda, N.H., 2007. The Laws of Emotion, Mahwah, NJ: Lawrence Erlbaum Associates, Inc. Gallagher, S. & Zahavi, D. 2008. The Phenomenological Mind: An Introduction to Philosophy of Mind and Cognitive Science. (London and New York, NY: Routledge, 2008). Heft, H. 2001. Ecological Psychology in Context: James Gibson, Roger Barker, and the Legacy of William James’ Radical Empiricism. New York: Taylor & Francis/Psychology Press.. Kaufer, S. & Chemero, A. 2015. Phenomenology: An Introduction. Cambridge, UK: Polity Press. Knoblich, G., Butterfill, S., & Sebanz, N. 2011. Psychological research on joint action: theory and data. In B. Ross (Ed.) The Psychology of Learning and Motivation Vol. 54. Burlington MA,: Elsevier Academic Press, pp.59-101. Reed, E. 1996. Encountering the world: Toward an ecological psychology. New York, NY: Oxford University Press. Richardson, M.J., Marsh, K.L., & Baron, R.M. 2007. Judging and actualising intrapersonal and interpersonal affordances. Journal of Experimental Psychology: Human Perception and Performance. 33: 845-859. Rietveld, E., & Kiverstein, J. (2014). A rich landscape of affordances. Ecological Psychology, 26(4), 325–352. doi:10.1080/10407413.2014.958035. Rietveld, E. (2008). Situated Normativity: The Normative Aspect of Embodied Cognition in Unreflective Action. Mind. Tollefsen, D. & Dale, R. 2011. Naturalising joint action: a process-based approach. Philosophical Psychology 25 (3): 385-407. Tomasello, M. 2019. Becoming Human: A Theory of Ontogeny. Cambridge, MA: Harvard University Press. Van Den Herik, J. & Rietveld, E. 2020. Reflective situated normativity. Philosophical Studies Van Dijk, L. & Rietveld, E. 2017. Foregrounding sociomaterial practice in our understanding of affordances: the skilled intentionality framework.

# Peer review: Reviewer response for version 1

**DOI:** 10.21956/openreseurope.14481.r26954

**Published:** 2021-07-08

**Authors:** Glenda Satne

**Affiliations:** 1 Philosophy, School of Humanities and Social Inquiry, Faculty of Arts, Social Sciences and Humanities, University of Wollongong, Wollongong, Australia

*“Skilled we-intentionality: Situating joint action in the living Environment”* argues that the literature on joint action, mainly devoted to understanding different activities in which two or more individuals act together, rather than alone or in parallel, has so far underplayed the role of environmental affordances in accounting for joint activities. To address this omission, the paper proposes a development of the already existing framework of skilled intentionality to study cases of we-intentionality in which individuals are engaged with others in joint activities.

 The paper is a good contribution to the literature and develops an interesting account of skilled action that incorporates the key role of the living environment in understanding joint action. Yet, the main claims in the paper are not entirely clear, and some of the key concepts proposed in the paper are ambiguous (in particular, that of ‘grip’ and ‘shared relevance’). This is because the paper is covering a variety of cases of joint activity that seem to work differently. Consequently, the key concepts put forward in the paper appear to apply differently to different cases of joint activity, if they apply at all. The paper needs to state its ambitions and main aim more clearly, as well as refine some of its central claims and conclusions. In what follows, I substantiate these points in more detail, while also commenting on the claims in the paper that invite clarification or expansion, for doing so can improve an already interesting and worthwhile paper.

**1.The first observation concerns the key concepts advanced in the paper.**

 In the following passage we are given a general characterization of the view:

 “People coordinate and join forces in acting together by responding to affordances of **shared relevance** in the living environment. When an affordance is **of shared relevance** to two or more individuals, these agents will then be ready to act together, to cooperate with each other and to pool their individual abilities, so as to **improve grip **on the overall situation in which the affordances are nested.” (p.7, my emphasis)

 According to this characterization, joint action coordination takes place when the environment offers the opportunity/invitation for individuals to act together. This is, when affordances are of ‘shared relevance’ for the individuals (ready to be) involved in the action.  ‘Shared relevance’ is defined in terms of ‘improvement of grip”, and ‘improvement of grip’ for a group of agents is defined in analogy with the individual case, that is, in terms of the reduction of disattunement in the agent-environment system **:**

**“**Why should it be that individuals sometimes find themselves ready to join forces and collaborate in responding to affordances of shared relevance? **The answer to this question is just the same as the answer we would give in the case of skilled intentionality for the actions of individuals. **Affordances stand out as **relevant **for an individual agent because of **disattunement in the agent-environment system, which the individual then acts to reduce.** […].” (p.10, my emphasis)

 Coordination is then characterized as a form of “interpersonal synergy” (pp. 7-8), in terms of which “improvement of grip” is jointly obtained. Thus, circumstances in which affordances stand out as of ‘shared relevance’ are those in which “the **only way** to improve grip is by forming an interpersonal synergy with other individuals, like in the example of the two-handed saw.” (p.10, my emphasis).

 It is important to note that the authors define a situation where there is shared relevance of affordances when “the only” way of improving grip is by forming an interpersonal synergy. Yet this description applies to certain forms of shared activity and not others.

 Some of the examples the authors give such as moving a heavy piano together down the stairs (p.8), or joining another agent to use a two-handed saw to cut through a tree (p.7), are cases where the definitions just discussed seem to apply cogently. Yet, in many cases of shared activity, there is no clear ‘grip’ improvement that is taking place. For example, imagine a crowd in a stadium joining in a ‘Mexican wave’ to celebrate the victory of their soccer team and someone sitting in the same stand as these winning team supporters, but who is not a supporter of that team and does not join in the wave. Later, her own team scores a goal and she celebrates by singing a song that the supporters of her team are singing at the other side of the stadium. How should we think of her not joining the wave in terms of the view proposed? Should we say that she is not reducing the disattunment in the agent-environment system by not joining in or should we say the opposite? What about the singing in the other side of the field? There is no obvious explanation in terms of disattunement here, for there seem to be different dimensions of explanation involved. The person’s relation to her own team, and her determination to act as a member of that team, might say more about her motivations and actions than her bodily situation in the current context. 

 Other sets of cases that seem very different from the two-handed saw are cases that involve acting with others, but that are not cases of bodily interaction. For example, we all paint the house together, but we came to paint on different days with explicit instructions distributed to us in advance. In this case, it seems that we need other elements apart from interpersonal synergy to explain coordination between agents. Though, in this case, the environment plays a key role, there is much more to be said about how these relations work - between agents, between agents and environment, in relation to plans, etc. - compared to the double hand-saw case.

 Another interesting kind of case is the following: I am about to cross the street and I see an old lady struggling to step down the sidewalk. I step in to help her. Maybe I do not clearly decide in the sense of making a conscious decision to help her, rather I jump in and help her cross the street. This action of mine arguably reduces my attunement with the environment for I am now part of a dyad with the old lady, and we, crossing together the street, are much clumsier and slower as a joint agential system that I would be if crossing alone (though, arguably, she is doing better in this joint action scenario than alone). It is not clear what the affordances of shared relevance are in this case, for I do not improve my grip on the situation by joining her, though when we are in fact crossing the street some degree of reduction of disattunment might help explain - in part - our bodily coordination. It may be argued that, in this case, it is my values and the prevailing social norms by which I orient my action that explain what I am doing and why I am doing it, and perhaps even how do it (since I am gentle, I wait longer than I would with a younger person, I do this because of respect and kindness, even if perhaps this is not the  most efficient way for us to cross the street (could I carry her in my arms instead?)). The authors come close to this sort of consideration when they say:

 “Affordances more generally stand out from the landscape as being of shared relevance to two or more individuals because of the socio-material practice of which the individuals are members (There is something that the individuals care about because of the practices in which they take part, and they are ready to join forces with relevant affordances and act together based on what matters to them as members of a practice.)” (p.9)

 This might be true. Incidentally this explanation seems to fit better not only with my three own cases here, but also with their own examples of the ‘silent carriage in the train’ (p.7) and those of ‘shopping for a home-renovation’ (p.8)  and ‘hunting for berries together’ (p.9), in which a shared activity is explain in terms of other shared activities and practices. But it is to be noted how these examples, as much as mine above, are hardly explainable in terms of the notions of improvement of grip and reducing disattunement in the agent-environment system, as defined in the paper.

 The disanalogy here runs deep, for if we were to keep the same terminology to explain how individuals are motivated to act and coordinate in all these cases, the terms would become highly metaphorical. Can my sitting silent in a silent carriage of a train be completely explained in terms of improvement of grip, independently of my aim of respecting the social norm of ‘keeping quiet’ that prevails in that setting? Can we explain Mexican waves or helping an old lady cross the street as agents’ ways of improving their grip in the agent-environment system or do we need in addition, and crucially, reference to the agents’ intentions, values or group memberships? These are cases in which socio-cultural norms and values in place partly explain what the agents are doing and why. In this, these cases are quite different than handling a piano together or a double-handed saw. In the latter, there is a clear bodily sense in which the action involves two agents, and its success requires bodily coordination that does indeed improve grip in the agents-environment system ­– the interpersonal synergy the authors speak about. Moreover, these same cases seem explainable in terms of coordination, independently of any further aims that the agents may have, except those that the coordination brings about, i.e. cutting the tree or moving the piano down the stairs. In the examples I provided though, whereas in some cases there is bodily coordination (e.g., the stadium wave; helping an old lady cross the street), this is either not a straightforward case of improving grip, and/or the motivations of the agents are not completely explainable independently of further values, intentions and aims. For these kinds of cases, we cannot explain what the affordances of shared relevance are merely in terms of improvement of grip, where this is understood in terms of reduction of disattunment of the agent-environment system by the sole means of forming interpersonal synergies. To explain all these cases in that way would turn terms like “relevance”, “disattunement”, and “grip” highly metaphorical and vague, for they would explain bodily coordination and sparse time-space coordination, cases where there is face-to-face interaction and cases where agents are anonymous to each other, cases of joint action motivated by group membership, and cases motivated by emotional contagion, etc., in exactly in the same way. It looks as if we do need more nuances here.

**2. Another key issue that results from the different cases that the authors aim to explain by the same means, is that depending on what case we focus on, the dialectics with the current accounts of joint action changes.**

 In p.9, the authors claim:

 “The agents that participate in a joint action **do not need to have predefined intentions or goals that specify who is to do what and when, in order to coordinate with each other**. Multiple agents **can have control over an action the states of action readiness self-organise to form interpersonal synergies in response to affordances of shared relevance**.”

 While this is true in the case of the two-handed saw, it is not true of the case of helping the old lady, or in their own examples of “hunting for berries” or “shopping for a home renovation”. In these cases, it seems that we need further explanation of what the agents are doing and those involve reference to something more: some goals, intentions, values, or social norms that the agents share. In this respect, these cases are sufficiently different from cases which can be understood in terms of “interpersonal synergies in response of affordances of shared relevance”.

*(NB: Some authors have attempted systematic characterizations and classifications of the different cases of joint action (see e.g. Knoblich et al. 2011 and Satne 2020a). According to these classifications, some joint/shared activities involve shared planes and/or shared intentions while others can be explained without making reference to any prior intentions, goals or plans (see Satne 2020b and Butterfill 2012) for alternative accounts of how to characterize each case). The latter are described as forms of “emergent coordination” (Knoblich et al. 2011), “minimalist” forms of joint action, (Butterfill 2016) or “basic joint intentional activities” (Satne 2020a, b; Fiebich & Gallagher 2013).)*

 This important difference between different kinds of joint activity has a direct impact on the claim that participants in joint action “do not need to have predefined intentions **that specify who is to do what and when, in order to coordinate with each other”. **(p.9). When we focus on cases of joint action that are not defined by previous intentions or goals, in which bodily coordination plays a key role, i.e. the two-handed saw, or moving a piano together up or down the stairs, the authors’ argument runs smoothly, and the claim that their account can do without attributing intentions to explain coordination is sound (for a similar claim see Satne 2020a and Knoblich 2011). Thus, for these cases the concept of ‘interpersonal synergy’ and that of affordances of ‘shared relevance’ do indeed explain the kind of bodily coordination between different agents that takes place and how agents might be motivated to participate in joint action without having any predetermined intentions or goals. But when we consider the larger set of cases that they use to illustrate the view, this simply does not follow. ‘Shopping for a home-renovation’, ‘being in the silent carriage’, ‘painting a house together at different times’, they all involve some pre-determined goals, intentions or norms in terms of which who takes part in a joint action and when are determined. Even if the authors would want to resist this claim, much more argument would be needed. Crucially this depends on how one understands what intentions, goals, or norms are, and how they work. Thus, much more argument would be required to substantiate the claim that “interpersonal synergies in response of affordances of shared relevance” could explain such cases - and every possible case of shared activity - in the absence of any intentions, plans, goals, or norms.

 In sum, there are key issues that the authors need to address to clarify their proposal and make it cogent. Depending on how they address these, they could follow two alternative paths:

1. circumscribe the focus of the paper to explain bodily coordination in joint action. This can include cases of ‘emergent coordination’ (Knoblich et al. 2011), that is, cases of bodily coordination where there are no previous plans or preceding intentions or goals. This study can also include as one kind of case of bodily coordination, instances of acting together that involve bodily coordination but also further aims or goals (e.g. helping the old lady, riding on the silent carriage), in as far as it is clearly stated that the paper’s aim is not to explain all aspects of these joint actions, but only bodily coordination in relation to the environment, and in this the explanation works as a complement rather that a rival to other views. It is clear from the paper’s arguments how the proposal applies to such cases, explaining them in terms of interpersonal synergies, shared relevance of affordances, and improvement of grip.
2. aim at an encompassing account, that covers all different cases of skilled we-intentionality. If the authors were to follow the second route, they would need to have a different focus for the paper and make correlative adjustments in the main arguments. This might require that some of characterisations of joint action in terms of ‘shared relevance’ and ‘improvement of grip’ as applying to all cases, be dropped, for broadening the scope of these terms too much, risks turning them vague or empty. A good strategy here would be to analyse the different kinds of cases of joint action in more detail, explaining in what sense different cases of joint action can be accounted for with those same concepts, or nuances thereof, and in what sense any of these do involve any goals or intentions on the part of agents, that specify further aims, intentions, or goals that the agents aim to fulfil by acting in that way.

While both alternatives require refinements in the paper’s claims, option (1) seems more feasible in the light of the materials discussed in the paper. Furthermore, as explained in more detail in Section 3, the arguments against rival theories of joint action are straightforward in this case, while they are not if the scope is broadened to cover all sorts of different cases. In this version of the paper, the aim of broadening the scope and applying the skilled intentionality tools to all cases of joint action could be mentioned but left for detailed discussion in a different paper (or several), entirely devoted to making those points.

**3. Accounts of joint action have several different aims, some of which the account presented by the authors addresses, while others not.**

 It would be good for the authors to clarify what exactly their account aims to do with regards to these different debates and be explicit about it.

 a) One of the main aims of theories of joint action is to explain what differentiates joint action and an action done by two individuals but in parallel.

 The paper does not explicitly address this point. Yet, it might be that some ideas in the paper - e.g. interpersonal synergy - could account for this difference at least for cases of bodily coordination. However, if the paper aims to cover other cases, such as the ‘painting the house’ where joint action is remote in space and time, more argument would be required to support this claim.

 b) Another aim that many views on joint action have is to position themselves on whether we can give an account of joint action in terms of individual states plus other conditions, or not. This amounts to asking whether ‘jointness’ in ‘joint action’ can be accounted for in terms that do not presuppose jointness/sharedness or not. Reductionist accounts (e.g. Bratman, Ludwig) claim that we can, antireductionist accounts (e.g. Gilbert, Searle, Schmid) deny it.

 The paper only briefly refers to this debate, in its discussion of Searle’s view that mainly concerns, as with Bratman’s, (c) below (i.e.  whether inter-agential coordination can/should be explained in terms intentions). Now, one can wonder whether the ‘Skilled-intentionality’ view, in its own terms, is anti-reductionist with respect to its ‘jointness’.

 The position is not clear in the paper, and there are no arguments explicitly addressing this question. On the one hand, in as far as ‘shared relevance’ and ‘improvement of grip’ explain joint action in terms of reducing disattunment of the agents-environment systems, the view could be seen as reductionist. Yet, this changes when we consider the cases that require framing in terms of further shared activities, like the case of shopping for a home-renovation. In those cases, it seems that ‘shared activities’ – decorating the house ­– explain ‘shared relevance’ – looking at this particular tool in the DIY-store­ – and these shared activities in turn are explained by ‘a history of interactions’ – the couple plans to renovate their house, etc. In this line of argument, ‘sharedness’ might be seen as a primitive concept, for it leads to other forms of sharedness that together might be thought to form an explanatory circle.

 Thus, here again we find an internal tension and an ambiguity in the view, for different cases seem to require different accounts. Even if one thought, as the paper also claims, that affordances are always nested in other affordances, and thus that all cases are like cases of the second kind, this requires further explaining. In any case, whether this is the authors’ view is speculative, for this issue is not explicitly discussed in the paper.

 c) Some views on joint action attempt to explain ‘coordination in joint action’ and discuss what elements are required to explain this, e.g. whether intentions or plans are required.

 The paper gives an account of how coordination might work in many cases, relying on affordances provided by the living environment without the need of agents having plans and following up on them. In this, the paper is successful. In this respect, the proposal is indeed different from Bratman’s, as the paper claims. Yet, it is important that the paper clarifies explicitly that these are not rival accounts, as Bratman states explicitly that his view is meant only as offering sufficient conditions – not necessary ones ­– for shared intentional activity, and the authors do not disagree that there might be cases of joint action where there are such explicit plans. In p.4, they explicitly say this: “The account of interlocking intentions Bratman provides is perhaps well-suited to account for the negotiations that might unfold between people when they plan to perform an action together (e.g., Bratman, 2014). However, it does not explain how multiple agents succeed in putting the plans they have made into effect, coordinating what they are doing when they act together in a particular situation.” While this last point might be true, Bratman does not claim to be explaining this, for Bratmanian joint action might involve no bodily coordination at all. A Bratmanian case of joint action may be like the example of agents coming to paint the house at different times, mentioned above, where there is no face-to-face interaction, and agents have explicit plans and common knowledge of those. As long as there are some cases in which there are individual intentions and common plans, Bratman’s account is not challenged.

 In any case, beyond Bratman, the paper shows how intentions need not be considered necessary for inter-agential action coordination to occur. Yet the authors claim that they are offering an account of joint action or joint activity that does away with intentions or goals altogether (see e.g. “Our aim […]is to show how the work that shared goals and intentions are asked to do in philosophical accounts of joint action can instead be performed by affordances of shared relevance.”, p.5). This - I think - is not shown in the paper. While the arguments might show this is the case for spontaneous actions that involve bodily coordination between individuals, it is not clear from the considerations in the paper that this is so in all cases that the paper mentions.

 d) Finally, some accounts are interested in arguing that the “sharedness” of joint action is dependent on the presence of a particular subject, a first-person plural subject or a ‘we’, where ‘we’ is understood as a plural agent not reducible to a mere sum of individuals (see Schmid and Gilbert for paradigmatic views). 

 The paper does not take a position on this aspect of the discussion. This is in part due to the fact that the paper does not clarify what is meant by ‘we’ in “we-intentionality”.  (See (4) below for some remarks on this issue.)

 In sum, in relation to the current scholarly discussion on joint action, the account presented in the paper clearly addresses (c) by making the case that affordances in the living environment, that are of shared relevance for the individuals, can account for inter-agential action coordination in the absence of intentions directed at common plans (or subplans that fit a shared plan). As said, the paper’s criticism of the literature on this issue is partly successful, that is, in as far as it focuses on the explanation of action coordination cases that do not require a further specification of the agents’ activities in terms of further joint aims, shared activities or shared goals of the agents. Yet it is not clear that the view succeeds as a general alternative to Searle’s or Bratman’s accounts when all cases of shared activity are included. As said above, this will require more and different kinds of argument.

 Thus, while the paper could succeed in explaining coordination in action, it should clearly state its scope and aim as explaining how coordination is possible without intentions, rather than claiming that this is the case for all possible cases of shared activity, and attempting to cover a wide range of very different cases with the concepts of “shared relevance” and “improvement of grip”. In this regard, I recommend revision of the way the paper describes its aim, and correlative adjustments in the description of its conclusion, as well as examples, in line with (1) in (2) above. The paper would be best presented as mainly centred around (c), and addressing (a) and (b) in as far as they are corollaries of the analysis of the relevant cases, such as the double-handed saw. These corollaries should be drawn as explicitly addressing these questions for the relevant set of cases, i.e. those cases where an interpersonal synergy is developed as a way of improving grip for the agents involved.

 In the case of (b), as said above, the authors might endorse a form of explanatory reductionism that explains joint action in terms of shared relevance, and that, in turn, in terms of improvement of grip. Alternatively, they may endorse a form of antireductionism, where ‘sharedness’ is seen as a primitive concept that leads to other forms of sharedness that together form an explanatory circle. It should be noted though, that to follow the second line of reasoning has consequences for the overall argument and aim of the paper. For according to such anti-reductionism, there is no explanation of ‘joint’ in joint action ((a) above), and a different answer to (c) follows as well. For, even if the account explains coordination in joint action, it is not obvious that an antireductionism of this kind does away with intentions altogether, since those might be part of the further framework in which action coordination is nested, and thus agents’ intentions might be part of what is required to explain how action coordination works *in toto*. Thus, for this kind of anti-reductionist view, it won’t be (obviously) true that agents “do not need to have predefined intentions or goals that specify who is to do what and when, in order to coordinate with each other” even in the cases where interpersonal synergy builds from bodily coordination (e.g. the two-handed saw case). While in this respect the view might do away with intentions that explain *the steps* in the coordination involved in carrying out an activity (in this the paper might still complement Bratman’s and Searle’s accounts), at least in cases where interpersonal bodily synergies are involved, this is not to do away with any intentions/goals specifying the shared activity as such. These joint actions might be seen as embedded in other shared activities that involve more mediated outcomes, and are carried out by agents acting in virtue of complex shared values or norms, which in turn specify goals or intentions for the agents involved.

**4 A final concern relates to the use of the term “we-intentionality” in the paper.**

 Not only do different authors mean quite different things by that term, but the paper does not address the key questions that surround that concept in the relevant literature. One set of issues relates to what is meant by ‘we’ in “we-intentionality”, and another set of issues to what is meant by “intentionality”. Both are a matter of controversy.

 The authors characterise “we-intentionality” in the following terms: “what individuals do when they engage in group ways of thinking, feeling and acting” (see abstract, and cfr. p.3). Yet the focus of the paper is much narrower. It focuses on how the environment supports joint action and has much less or almost anything to say about shared feelings (there is a mention that affectivity is always involved in shared activity) or shared thoughts (which do not figure at all). The same applies to the reference to groups in the previous quote. The paper does not discuss in any detail how groups behave, beyond the case of dyads interacting. So, I recommend narrowing the focus to the main target of the paper, with an exclusive and explicit focus on joint action of dyads - and especially coordinating activities within those, perhaps within the larger topic of we-intentionality. The use of this term, if kept, will minimally require an explanatory footnote, noting some of the relevant discussions and some further references to others in the field using the term in related ways.

If any, are all the source data and materials underlying the results available?

No source data required

Is the work original in terms of material and argument?

Yes

Is the work clearly and cogently presented?

Partly

Is the argument persuasive and supported by evidence?

Partly

Does the research article contribute to the cultural, historical, social understanding of the field?

Yes

Does it sufficiently engage with relevant methodologies and secondary literature on the topic?

Yes

Reviewer Expertise:

NA

I confirm that I have read this submission and believe that I have an appropriate level of expertise to confirm that it is of an acceptable scientific standard, however I have significant reservations, as outlined above.

## References

[1] : Joint Action and Development. *The Philosophical Quarterly* .2012;62(246) : 10.1111/j.1467-9213.2011.00005.x 23-47 10.1111/j.1467-9213.2011.00005.x

[2] : Joint action: a minimalist approach. *The Routledge Handbook of Philosophy of the Social Mind* .2016; 10.4324/9781315530178-30 10.4324/9781315530178-30

[3] : 'Joint attention in joint action'. *Philosophical Psychology* .2013;26(4) : 10.1080/09515089.2012.690176 571-587 10.1080/09515089.2012.690176

[4] : Psychological Research on Joint Action: Theory and Data. *Psychology of Learning and Motivation, Academic Press* .2011;54: 10.1016/B978-0-12-385527-5.00003-6 59-101 10.1016/B978-0-12-385527-5.00003-6

[5] : Practical knowledge and shared agency: pluralizing the Anscombean view. *Inquiry* .2020; 10.1080/0020174X.2020.1837236 1-28 10.1080/0020174X.2020.1837236

[6] : Understanding others by doing things together: an enactive account. *Synthese* .2021;198(S1) : 10.1007/s11229-020-02692-2 507-528 10.1007/s11229-020-02692-2

# Response

**Published:** 2021-09-08

**Authors:** Julian Kiverstein

**Affiliations:** Amsterdam University Medical Research, Netherlands Antilles

**The aims of our paper** *The authors need… to clarify their proposal and make it cogent. Depending on how they address these, they could follow two alternative paths:*

1. *circumscribe the focus of the paper to explain bodily coordination in joint action.*
2. *aim at an encompassing account, that covers all different cases of skilled we-intentionality. This might require that some of characterisations of joint action in terms of ‘shared relevance’ and ‘improvement of grip’ as applying to all cases, be dropped, for broadening the scope of these terms too much, risks turning them vague or empty.*

 
**Author’s reply: **Our aim was to provide a framework for understanding we-intentionality –  understood as what individuals do when they think, act and feel as a member of a group – *in terms of skilled intentionality*. We therefore resisted the more modest framing suggested by Prof Satne under option 1. We define ‘skilled intentionality’ as the selective responsiveness to multiple relevant affordances at the same time. The definition of we-intentionality we propose is intended to be maximally broad, covering not only cases of joint action but also group ways of thinking, acting and feeling that are often discussed under the heading of collective intentionality. Thus it would be underselling the ambitions of the paper to present our account as a theory of bodily coordination in joint action (option 1 above). To adopt such a framing for the paper would suggest that skilled intentionality is primarily a theory of perception and action rather than providing a conceptual framework for understanding cognition across the board (so-called ‘higher’ cognition’ (sapience) as well as ‘lower cognition’ (sentience)). We have added a few sentences to clarify the programmatic aims of our paper to apply the skilled intentionality framework to we-intentionality. We do not claim to have provided a fully worked out account of we-intentionality that can be applied to all possible cases. Our aim is to instead provide some useful conceptual tools that will enable this kind of work to be done by ourselves, and others in the future.   

**The explanatory limits of the Skilled Intentionality Framework** *(In) many cases of shared activity, there is no clear ‘grip’ improvement that is taking place. For example, imagine a crowd in a stadium joining in a ‘Mexican wave’ to celebrate the victory of their soccer team and someone sitting in the same stand as these winning team supporters, but who is not a supporter of that team and does not join in the wave. Later, her own team scores a goal and she celebrates by singing a song that the supporters of her team are singing at the other side of the stadium. How should we think of her not joining the wave in terms of the view proposed? Another interesting kind of case is the following: I am about to cross the street and I see an old lady struggling to step down the sidewalk. I step in to help her. It is not clear what the affordances of shared relevance are in this case. The disanalogy here runs deep, for if we were to keep the same terminology to explain how individuals are motivated to act and coordinate in all these cases, the terms would become highly metaphorical. Can we explain Mexican waves or helping an old lady cross the street as agents’ ways of improving their grip in the agent-environment system or do we need in addition, and crucially, reference to the agents’ intentions, values or group memberships? These are cases in which socio-cultural norms and values in place partly explain what the agents are doing and why. In this, these cases are quite different than handling a piano together or a double-handed saw. In the examples I provided though, whereas in some cases there is bodily coordination (e.g., the stadium wave; helping an old lady cross the street), this is either not a straightforward case of improving grip, and/or the motivations of the agents are not completely explainable independently of further values, intentions and aims. For these kinds of cases, we cannot explain what the affordances of shared relevance are merely in terms of improvement of grip, where this is understood in terms of reduction of disattunment of the agent-environment system by the sole means of forming interpersonal synergies.*

**Author’s reply:** We are grateful to Prof Satne for this challenge, and for presenting us with these intriguing apparent counterexamples. We think we can accommodate the cases she has described by better foregrounding the role that situated normativity plays in tending towards an improved grip. ‘Situated normativity’ is the term we have used in previous work to refer to the ability of skilled individuals to distinguish better from worse, adequate from inadequate, appropriate from inappropriate, or correct from incorrect in the context of a particular situation (Rietveld 2008). Improvements in grip are sensed by the individual based on situated normativity, also, importantly, in cases of so-called “higher” cognition or what we have called *reflective* situated normativity (van den Herik & Rietveld 2021). Reflective situated normativity is a form of normativity that relies on linguistic practices and involve explicit articulation of rules. If I tell my children: “No phones at the dinner table!”, this is an example of reflective situated normativity. The articulated rule attracts attention to and puts on display a pattern of behaviour as inappropriate. It can therefore be characterised as reflective. Crucially the ability to make normative distinctions between better or worse, adequate or inadequate in acting skilfully is one that human agents develop and cultivate by taking part in social and cultural practices. Situated normativity is always in play whenever the individual acts to improve grip on the field of relevant affordances. We would not agree that the notion of grip requires supplementing with “further values, intentions and aims” as Prof Satne suggests. We would instead propose that this explanatory work can instead be achieved by reflecting on the role of situated normativity in regulating the agent-environment dynamics such that the agent tends towards improved grip in acting skilfully. In our revisions, we show how this might work in the case of helping the old lady to cross the street (in footnote 12, p.17). Grip increases by helping her because one would experience what we call ‘discontent’ (an affective tension) were one to leave her to make her way across on her own. Acting to help her thereby, on the other hand, reduces the feeling of discontent that is experienced as long as she appears to be at risk. A similar line of argument could be made for the example of the Mexican wave, though we did not add this example to the paper. The person that does not participate in the Mexican wave refrains because her taking part would feel wrong. The invitation to join in from others elicits tension, which she reduces by remaining in her seat. Thus, her declining to take part is a way for her to improve grip.      

**The explanatory role of predefined goals and intentions** *In the case of helping the old lady, or in their own examples of “hunting for berries” or “shopping for a home renovation”… it seems that we need further explanation of what the agents are doing and those involve reference to something more: some goals, intentions, values, or social norms that the agents share. ‘Shopping for a home-renovation’, ‘being in the silent carriage’, ‘painting a house together at different times’, they all involve some pre-determined goals, intentions or norms in terms of which who takes part in a joint action and when are determined. Even if the authors would want to resist this claim, much more argument would be needed.*

**Author’s reply:** As already noted, we fully agree that normativity is crucial and that is why we coined (and extensively argued for) the notions of unreflective situated normativity (Rietveld 2008) and more recently reflective situated normativity (van den Herik & Rietveld 2021), see our previous reply.  However, as we note on p.10 of our paper, we agree with Lucy Suchman that the explanatory appeal to pre-determined goals or intentions, which is very common in the philosophy of cognitive science, explains very little. How an agent constrains and directs their action in the light of this goal or intention remains something of a mystery. We suggest the explanatory work of these constructs can be taken over instead by the self-organising dynamics of the agent-environment system. We have developed the Skilled Intentionality Framework as a conceptual framework for a post-cognitivist cognitive science that aims to take concepts such as goal and intention and provide tools for making sense of these concepts in the terms of ecological-enactive cognitive science. Now clearly it would be a problem for us if the conceptual tools we were proposing somehow had to rely on goals and intentions to do their explanatory work. We would face an objection of tacitly appealing to the very concepts we seek to render intelligible. However, so far as we can tell our account is not vulnerable to such an objection. For instance, we do not need to appeal to plans, intentions or goals to explain why the couple went shopping for DIY materials. We instead appeal to what we call ‘large-scale affordances’ of renovating the house  - small-scale affordances such as all of the activities that contribute to our visiting the DIY shop for materials weave together over time because of our responsiveness to the large scale affordance of renovating the house (see also our ethnography of a shopping trip to a carpet warehouse in van Dijk & Rietveld, 2018: p.12-13). Do we not need to appeal to the goal of renovating their house to explain why the couple undertake such a shopping trip in the first place? Our proposal is to have the work of this overarching plan taken over by tending towards an improved grip on a large-scale affordance. We connect this self-organising dynamic to free-energy minimisation in other work (see e.g. Bruineberg & Rietveld 2019). Thus, we are able to provide some empirical support for this proposal based on state-of-the-art work in theoretical neurobiology. SIF has the explanatory resources to unpack how to make sense of such a self-organising process of future-directed action without having recourse to plans, goals and intentions.  

**Doing justice to the different projects in the literature on we-intentionality** *It would be good for the authors to clarify what exactly their account aims to do with regards to these different debates and be explicit about it.*

1. *what differentiates joint action and an action done by two individuals but in parallel?*

**Author’s reply:** This question is addressed in the section headed Interpersonal synergies. Interpersonal synergies make possible a pooling of individual abilities that make joint actions possible that would not be possible for individuals acting in parallel.

1. *Can ‘jointness’ in ‘joint action’ be accounted for in terms that do not presuppose jointness/sharedness or not?*

**Author’s reply:** Our aim is to have (1) affordances of shared relevance and (2) correlative abilities acquired by taking part in sociomaterial practices take over the explanatory work that interlocking intentions are asked to do in, for example, Bratman’s account of action coordination. The sharing of affordances can be understood in two ways in our account. First, the landscape of affordances is defined in relation to a *shared* form of life (and that can be a sociomaterial practice in the case of human beings). The affordances that the landscape makes available have formed through patterns of practice that are constituted by the coordinated behaviour of multiple individuals. What groups, dyads and individuals share is that they are situated in the rich landscape of affordances. Moreover, the affordances the landscape makes available owe their continued existence to the actions of individuals that contribute to the continued availability of those affordances. We see this in the example of the silent carriage where the individual contributes to maintaining a sociocultural practice. Second, we account for joint action in terms of how interpersonal synergies form between individuals. Affordance related states of action readiness form in each of the individuals as they tend towards an improved grip on the environment (Bruineberg & Rietveld, 2019). Action readiness is a state of the whole body that is simultaneously behavioural and affective (Frijda 2007). What is different in cases of joint action is that the abilities that account for the formation of patterns of action readiness in individuals are pooled which has the consequence that affordances can stand out from the landscape as being of *shared* relevance.  

1. *Some views on joint action attempt to explain ‘coordination in joint action’ and discuss what elements are required to explain this, e.g. whether intentions or plans are required.*

**Author’s reply:** We addressed this objection above in our response to your first query about the scope of our argument. The aim of our framework is to account for the explanatory work that traditionally intentions and plans are supposed to do in terms of selective and simultaneous responsiveness to multiple relevant affordances, including large scale affordances.

1. *some accounts are interested in arguing that the “sharedness” of joint action is dependent on the presence of a particular subject, a first-person plural subject or a ‘we’, where ‘we’ is understood as a plural agent not reducible to a mere sum of individuals (see Schmid and Gilbert for paradigmatic views). *

**Author’s reply:** We do not directly address this question in our paper, but we have now added a footnote to explain the form that this question takes in our account. Something like this question comes when we consider whether the pooling of abilities that happens when interpersonal synergies form results in a group agent or not. If we say that individuals that are acting as parts of an interpersonal synergy form a group agent, this would imply an important difference between skilled intentionality and skilled *we*-intentionality. In the former the skilled agent is an individual, while in skilled we-intentionality the agent is a dyad or a larger group. We claim however that the difference is best understood in terms of timescales, and thus there is no need for us to invoke a group agent. Over long timescales, individuals are always coordinating with the multiple members of a sociomaterial practice. We saw this in the example of the silent train carriage. Over shorter time scales, this coordination requires the pooling of abilities in ways that lead to the formation of interpersonal synergies.

        

**References** Bratman, M.E. 1993. Shared intention. *Ethics *104: 97-113. Bruineberg, J. & Rietveld, E. 2019. What’s inside your head once you’ve figured out what your head is inside of. *Ecological Psychology *31 (3): 198-217.  Frijda, N.H., 2007. *The Laws of Emotion*, Mahwah, NJ: Lawrence Erlbaum Associates, Inc. Gallagher, S. & Zahavi, D. 2008. *The Phenomenological Mind: An Introduction to Philosophy of Mind and Cognitive Science.* (London and New York, NY: Routledge, 2008). Heft, H. 2001. *Ecological Psychology in Context: James Gibson, Roger Barker, and the Legacy of William James’ Radical Empiricism. *New York: Taylor & Francis/Psychology Press.. Kaufer, S. & Chemero, A. 2015. *Phenomenology: An Introduction.* Cambridge, UK: Polity Press. Knoblich, G., Butterfill, S., & Sebanz, N. 2011. Psychological research on joint action: theory and data. In B. Ross (Ed.) *The Psychology of Learning and Motivation *Vol. 54. Burlington MA,: Elsevier Academic Press, pp.59-101. Reed, E. 1996. *Encountering the world: Toward an ecological psychology*. New York, NY: Oxford University Press. Richardson, M.J., Marsh, K.L., & Baron, R.M. 2007. Judging and actualising intrapersonal and interpersonal affordances. *Journal of Experimental Psychology: Human Perception and Performance. *33: 845-859. Rietveld, E., & Kiverstein, J. (2014). A rich landscape of affordances. *Ecological Psychology*, *26*(4), 325–352. doi:10.1080/10407413.2014.958035. Rietveld, E. (2008). Situated Normativity: The Normative Aspect of Embodied Cognition in Unreflective Action. *Mind*. Tollefsen, D. & Dale, R. 2011. Naturalising joint action: a process-based approach. *Philosophical Psychology *25 (3): 385-407. Tomasello, M. 2019. *Becoming Human: A Theory of Ontogeny*. Cambridge, MA: Harvard University Press. Van Den Herik, J. & Rietveld, E. 2020. Reflective situated normativity. *Philosophical Studies *Van Dijk, L. & Rietveld, E. 2017. Foregrounding sociomaterial practice in our understanding of affordances: the skilled intentionality framework.
